# Human NAIP/NLRC4 and NLRP3 inflammasomes detect *Salmonella* type III secretion system activities to restrict intracellular bacterial replication

**DOI:** 10.1371/journal.ppat.1009718

**Published:** 2022-01-24

**Authors:** Nawar Naseer, Marisa S. Egan, Valeria M. Reyes Ruiz, William P. Scott, Emma N. Hunter, Tabitha Demissie, Isabella Rauch, Igor E. Brodsky, Sunny Shin

**Affiliations:** 1 Department of Microbiology, University of Pennsylvania Perelman School of Medicine, Philadelphia, Pennsylvania; 2 Department of Molecular Microbiology and Immunology, Oregon Health & Science University, Portland, Oregon; 3 Department of Pathobiology, University of Pennsylvania School of Veterinary Medicine, Philadelphia, Pennsylvania; Children’s Hospital Boston, UNITED STATES

## Abstract

*Salmonella enterica* serovar Typhimurium is a Gram-negative pathogen that uses two distinct type III secretion systems (T3SSs), termed *Salmonella* pathogenicity island (SPI)-1 and SPI-2, to deliver virulence factors into the host cell. The SPI-1 T3SS enables *Salmonella* to invade host cells, while the SPI-2 T3SS facilitates *Salmonella*’s intracellular survival. In mice, a family of cytosolic immune sensors, including NAIP1, NAIP2, and NAIP5/6, recognizes the SPI-1 T3SS needle, inner rod, and flagellin proteins, respectively. Ligand recognition triggers assembly of the NAIP/NLRC4 inflammasome, which mediates caspase-1 activation, IL-1 family cytokine secretion, and pyroptosis of infected cells. In contrast to mice, humans encode a single NAIP that broadly recognizes all three ligands. The role of NAIP/NLRC4 or other inflammasomes during *Salmonella* infection of human macrophages is unclear. We find that although the NAIP/NLRC4 inflammasome is essential for detecting T3SS ligands in human macrophages, it is partially required for responses to infection, as *Salmonella* also activated the NLRP3 and CASP4/5 inflammasomes. Importantly, we demonstrate that combinatorial NAIP/NLRC4 and NLRP3 inflammasome activation restricts *Salmonella* replication in human macrophages. In contrast to SPI-1, the SPI-2 T3SS inner rod is not sensed by human or murine NAIPs, which is thought to allow *Salmonella* to evade host recognition and replicate intracellularly. Intriguingly, we find that human NAIP detects the SPI-2 T3SS needle protein. Critically, in the absence of both flagellin and the SPI-1 T3SS, the NAIP/NLRC4 inflammasome still controlled intracellular *Salmonella* burden. These findings reveal that recognition of *Salmonella* SPI-1 and SPI-2 T3SSs and engagement of both the NAIP/NLRC4 and NLRP3 inflammasomes control *Salmonella* infection in human macrophages.

## Author summary

*Salmonella enterica* serovar Typhimurium is a gastrointestinal bacterial pathogen that causes diarrheal disease and is a major cause of morbidity and mortality worldwide. *Salmonella* uses molecular syringe-like machines called type III secretion systems (T3SSs) to inject virulence factors into host cells. These T3SSs enable *Salmonella* to infect and survive within host cells such as macrophages. However, host cells contain a family of cytosolic immune receptors, termed NAIPs, that recognize T3SS and flagellin components. Upon detecting these components, NAIPs initiate formation of signaling complexes called inflammasomes. Inflammasomes activate host proteases called caspases that mount robust immune responses against the invading pathogen. While mice encode multiple NAIPs that have been extensively studied, much remains unknown about the role of the single human NAIP in host responses to *Salmonella*. We find that while NAIP is necessary to detect individual T3SS ligands, it is only partially required for inflammasome responses to *Salmonella* infection in human macrophages. We found that the NLRP3 and CASP4/5 inflammasomes are also activated, and the combination of NAIP- and NLRP3-mediated responses limits intracellular *Salmonella* replication in human macrophages. Our results demonstrate that human macrophages employ multiple inflammasomes to mount robust host defense against *Salmonella* infection.

## Introduction

*Salmonella enterica* serovar Typhimurium (referred to hereafter as *Salmonella*) is a Gram-negative bacterial pathogen that causes self-limiting gastroenteritis in immune-competent humans. Transmission of *Salmonella* typically occurs upon ingestion of contaminated food or water. Once inside the host, *Salmonella* uses specialized nanomachines known as type III secretion systems (T3SSs) to inject effectors into the host cell cytosol [1]. Subsequently, these effectors remodel host cellular processes to facilitate bacterial colonization and intracellular survival. Thus, *Salmonella*’s T3SSs enable the enteric pathogen to successfully colonize the intestinal tract and infect a variety of cell types, including intestinal epithelial cells (IECs) and macrophages [1]. Specifically, *Salmonella* uses its first T3SS, located on *Salmonella* pathogenicity island 1 (SPI-1), to invade host cells, and its second T3SS, located on a second pathogenicity island, SPI-2, to persist and replicate within host cells [2–10]. Numerous other Gram-negative bacterial pathogens also use these evolutionarily conserved T3SSs to colonize the host [11]. While T3SSs are required for these bacterial pathogens to cause disease, they also translocate flagellin [12] and structural components of the T3SS into the cytosol, thus enabling the host to detect the invading pathogen. Unlike effectors, which display significant diversity across bacterial species, structural components of the T3SS or the flagellar apparatus retain significant structural homology across Gram-negative bacteria [13–19]. Thus, these ligands serve as ideal targets for host immune sensors.

The mammalian innate immune system is armed with pattern recognition receptors (PRRs) that detect pathogens by recognizing pathogen-associated molecular patterns (PAMPs) [20,21]. A subfamily of cytosolic PRRs, known as NAIPs (the NLR [nucleotide-binding domain, leucine-rich repeat-containing] family, apoptosis inhibitory proteins), recognize the structurally related SPI-1 T3SS needle protein, SPI-1 T3SS inner rod protein, and flagellin, which are translocated into the host cell cytosol by the SPI-1 T3SS [12,14,22–25]. Mice have multiple NAIPs, each specific to a particular ligand: NAIP1 recognizes the T3SS needle protein, NAIP2 recognizes the T3SS inner rod protein, and NAIP5 and NAIP6 both recognize flagellin [22,23,26–29]. Upon sensing a ligand, NAIPs recruit the adaptor protein NLRC4 (nucleotide-binding domain, leucine-rich repeat-containing family, CARD domain-containing protein 4) to form multimeric signaling complexes called inflammasomes [30–32]. The NAIP/NLRC4 inflammasome then recruits and activates the cysteine protease caspase-1 [33–36]. Active caspase-1 cleaves downstream substrates, including pro-IL-1 and pro-IL-18, as well as the pore-forming protein gasdermin-D (GSDMD) [37–41]. Cleaved GSDMD creates pores in the host plasma membrane, leading to the release of proinflammatory cytokines and an inflammatory form of cell death known as pyroptosis, which effectively eliminates the infected cell.

Unlike mice, humans only express one functional NAIP [42,43]. In human macrophages, this single NAIP is sufficient to respond to the cytosolic delivery of bacterial flagellin as well as the SPI-1 T3SS inner rod (PrgJ) and needle (PrgI) proteins [44–46]. Interestingly, the SPI-2 T3SS inner rod protein (SsaI) fails to induce inflammasome activation in both murine and human macrophages [14,45], suggesting that the *Salmonella* SPI-2 T3SS evades NAIP detection to enable *Salmonella* replication within macrophages. The NAIP/NLRC4 inflammasome promotes control of intestinal *Salmonella* infection in mice by triggering pyroptosis and expulsion of infected intestinal epithelial cells [47,48]. Recently, both the NAIP/NLRC4 and NLRP3 (NLR pyrin domain-containing protein 3) inflammasomes have been shown to respond to *Salmonella* infection in human macrophages [49,50]. However, whether the NAIP/NLRC4 inflammasome recognizes the SPI-2 T3SS needle protein (SsaG), and whether the NAIP/NLRC4 and NLRP3 inflammasomes contribute to the restriction of *Salmonella* replication within human macrophages is unknown.

In this study, we found that while human macrophages require both NAIP and NLRC4 for inflammasome responses to T3SS ligands, NAIP and NLRC4 are only partially required for the inflammasome response during *Salmonella* infection. Rather, we found that infection of human macrophages with *Salmonella* grown under SPI-1-inducing conditions activates multiple inflammasomes, including NAIP/NLRC4, CASP4/5, and NLRP3. Importantly, both the NAIP/NLRC4 and NLRP3 inflammasomes played a functional role in restricting *Salmonella*’s intracellular replication, indicating that they contribute to host defense in a cell-intrinsic manner, as well as via release of inflammatory mediators. Finally, we found that the NAIP/NLRC4 inflammasome recognizes the SPI-2 T3SS needle protein SsaG, and that SPI-1 T3SS and flagellin-independent, NAIP/NLRC4-dependent recognition of *Salmonella* controls bacterial burden within human macrophages. Our studies uncover a multifaceted inflammasome response to *Salmonella* infection in human macrophages, and reveal that NAIP/NLRC4 inflammasome-dependent sensing of the SPI-2 T3SS promotes control of intracellular *Salmonella*. Collectively, these findings yield important insight into how human macrophages use inflammasomes to sense and respond to intracellular bacterial pathogens.

## Results

### NAIP and NLRC4 are necessary for inflammasome responses to T3SS ligands in human macrophages

In murine macrophages, multiple NAIPs are required for inflammasome responses to the *Salmonella* SPI-1 T3SS inner rod protein (PrgJ), the SPI-1 T3SS needle protein (PrgI), and flagellin [22,23,26–29]. In addition, the murine NAIPs and NLRC4 contribute to the inflammasome response during *in vivo Salmonella* infection [14,29]. In human macrophages, PrgJ, PrgI, and flagellin all activate the inflammasome, while the *Salmonella* SPI-2 inner rod protein (SsaI) does not [44,45]. Using siRNA-mediated silencing of *NAIP* in human macrophages, we have previously shown that human NAIP is important for maximal inflammasome responses to PrgJ and flagellin [45]. However, siRNA-mediated knockdown of *NAIP* did not completely abrogate inflammasome activation, either due to incomplete knockdown, or the potential contribution of other inflammasomes. Therefore, it remained unclear whether human NAIP or NLRC4 is absolutely required for inflammasome responses to these bacterial ligands or whether additional host sensors also mediate sensing of these ligands.

To test the requirement of the NAIP/NLRC4 inflammasome in human macrophages, we used the Clustered Regularly Interspersed Palindromic Repeat (CRISPR) system, in conjunction with the RNA-guided exonuclease Cas9, to disrupt the *NAIP* and *NLRC4* genes in the human monocytic cell line, THP-1 (S1A and S2A Figs). We selected one independent single cell clone of *NAIP*^*-/-*^ THP-1s (*NAIP*^*-/-*^ Clone #12) that exhibited reduced *NAIP* mRNA expression by qRT-PCR compared to WT THP-1s (S1C Fig). Sequence validation confirmed that this clone contained a deletion of 1 or 2 nucleotides in both *NAIP* alleles, resulting in premature stop codons (S1B Fig). We selected two independent single cell clones of *NLRC4*^*-/-*^ THP-1s (*NLRC4*^*-/-*^ Clone #4 and Clone #7), both of which showed complete loss of NLRC4 protein expression compared to WT THP-1s (S2D Fig). Both clones were sequence-validated and both alleles of each clone contained mutations that resulted in premature stop codons (S2B and S2C Fig). These sequence-validated *NAIP*^*-/-*^ and *NLRC4*^*-/-*^ THP-1 clones were used throughout this study.

**Fig 1:**
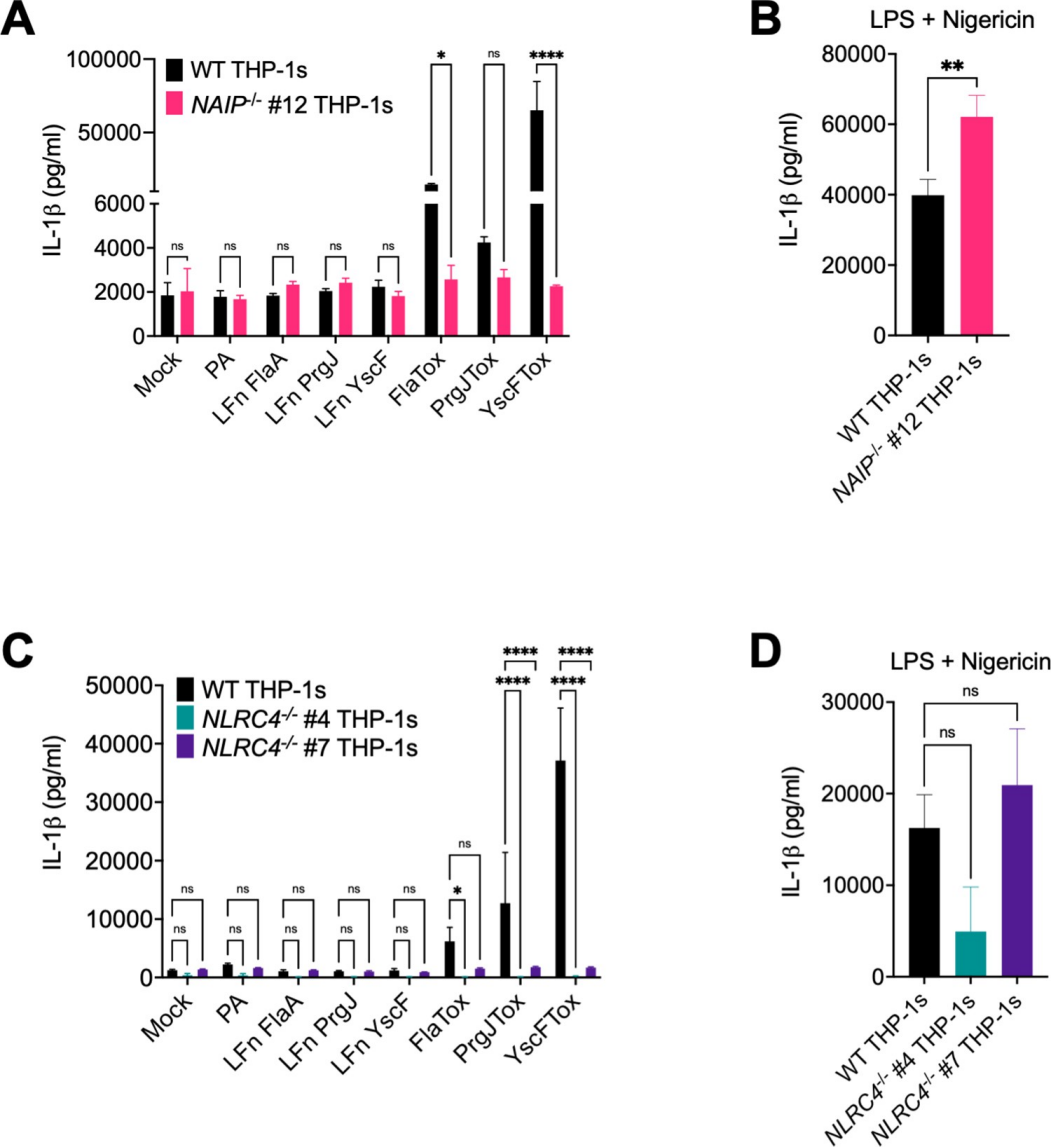
NAIP and NLRC4 are necessary for inflammasome responses to T3SS ligands in human macrophages. WT, *NAIP*^*-/-*^ clone #12, and two independent clones of *NLRC4*^*-/-*^ THP-1 monocyte-derived macrophages were primed with 100 ng/mL Pam3CSK4 for 16 hours. Cells were then treated with PBS (Mock), PA alone, LFnFlaA^310–475^ alone (LFnFlaA), LFnPrgJ alone, LFnYscF alone, PA+LFnFlaA^310–475^ (FlaTox), PA+LFnPrgJ (PrgJTox), or PA+LFnYscF (YscFTox) for 6 hours (A, C). As a control, cells were primed with 500 ng/mL LPS for 4 hours and treated with 10 μM nigericin for 6 hours (B, D). Release of IL-1β into the supernatant was measured by ELISA. ns–not significant, *p < 0.05, **p < 0.01, ****p < 0.0001 by Šídák’s multiple comparisons test (A), or by unpaired t-test (B), or by Dunnett’s multiple comparisons test (C, D). Error bars represent the standard deviation of triplicate wells from one experiment. Data shown are representative of at least three independent experiments.

To test if NAIP and NLRC4 are necessary for inflammasome responses to bacterial T3SS ligands, we used the *Bacillus anthracis* toxin system to deliver bacterial T3SS ligands into the cytosol of THP-1s [51], and assayed inflammasome responses in wild type (WT), *NAIP*^*-/-*^, and *NLRC4*^*-/-*^ THP-1 macrophages. The *B*. *anthracis* system contains two subunits: a protective antigen (PA) that creates a pore in the host endosomal membrane and a truncated lethal factor (LFn) that is delivered through the PA pore into the cytosol. Our T3SS ligands of interest are fused to the N-terminal domain of the *B*. *anthracis* LFn. When the LFn is added to eukaryotic cells in conjunction with PA (collectively referred to as Tox), the bacterial ligand is delivered directly into the host cell cytosol. Using this system, we delivered a truncated version of *Legionella* flagellin (FlaTox), the *Salmonella* SPI-1 T3SS inner rod protein (PrgJTox), and the *Burkholderia* T3SS needle protein (YscFTox) into THP-1s. We then measured the release of the inflammasome-dependent IL-1 family cytokines IL-1α, IL-1β, and IL-18 and cell death as markers of inflammasome activation. Cells left untreated (Mock) or treated with PA alone or LFn fused to the bacterial ligand alone released negligible levels of IL-1β, IL-18, and IL-1α and exhibited minimal cell death (Figs 1A, 1C, S3A–S3C and S4A–S4C). In agreement with previous findings [45], WT THP-1s treated with both the PA and LFn subunits exhibited robust inflammasome activation, and released substantial levels of IL-1β, IL-18, and IL-1α and exhibited considerable cytotoxicity (Figs 1A, 1C, S3A–S3C and S4A–S4C), indicating that robust inflammasome activation requires cytosolic delivery of the ligands. In contrast, both *NAIP*^*-/-*^ THP-1s and *NLRC4*^*-/-*^ THP-1s released negligible levels of inflammasome-dependent cytokines and did not undergo cell death when treated with FlaTox, PrgJTox, or YscFTox (Figs 1A, 1C, S3A–S3C and S4A–S4C). Importantly, the *NAIP*^*-/-*^ and *NLRC4*^*-/-*^ THP-1s released IL-1β at levels comparable to those released by WT THP-1s in response to the NLRP3 stimulus LPS + nigericin (Fig 1B and 1D), indicating that CRISPR/Cas9 editing was specific to the NAIP/NLRC4 inflammasome pathway [52]. In addition, release of the inflammasome-independent cytokine TNF-α was unaffected in *NAIP*^*-/-*^ or *NLRC4*^*-/-*^ THP-1s (S3D and S4D Figs). Consistent with our prior results [45] and in agreement with recent studies [49], these results collectively demonstrate that NAIP and NLRC4 are required for inflammasome activation in response to the T3SS inner rod, T3SS needle, and flagellin proteins in human macrophages.

### NAIP and NLRC4 are partially required for inflammasome activation during *Salmonella* infection of human macrophages

**Fig 2:**
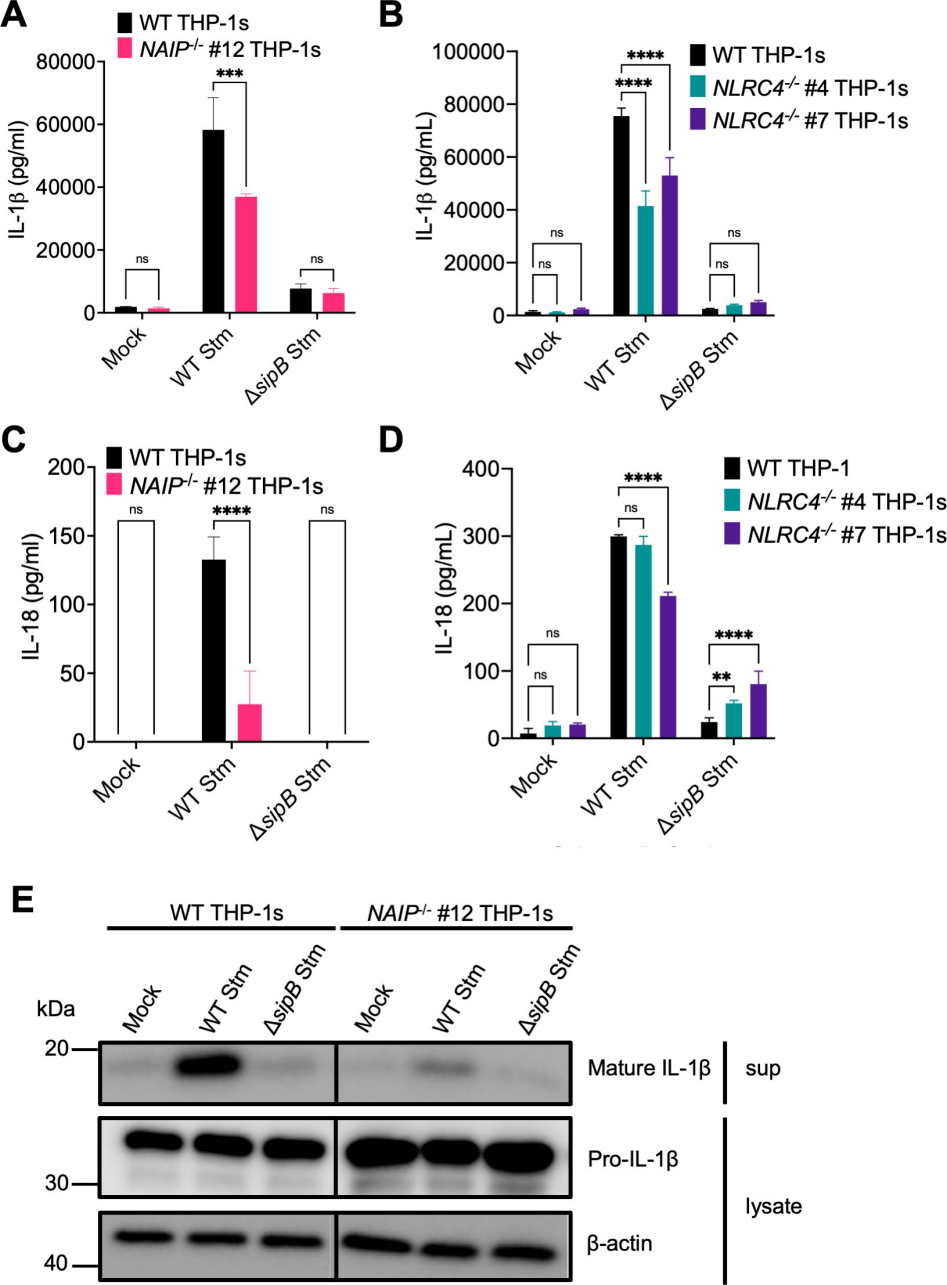
NAIP and NLRC4 are partially required for inflammasome activation during *Salmonella* infection in human macrophages. WT, *NAIP*^*-/-*^ clone #12, and two independent clones of *NLRC4*^*-/-*^ THP-1 monocyte-derived macrophages were primed with 100 ng/mL Pam3CSK4 for 16 hours. Cells were then infected with PBS (Mock), WT *S*. Typhimurium, or Δ*sipB S*. Typhimurium at an MOI = 20 for 6 hours. Release of IL-1β (A, B) and IL-18 (C, D) into the supernatant were measured by ELISA. Immunoblot analysis was performed on supernatants (sup) for mature IL-1β and on lysates for pro–IL-1β and β-actin as a loading control. ns–not significant, ***p < 0.001, ****p < 0.0001 by Šídák’s multiple comparisons test (A, C) or Dunnett’s multiple comparisons test (B, D). Error bars represent the standard deviation of triplicate wells from one experiment. Data shown are representative of at least three independent experiments.

Human macrophages undergo SPI-1 T3SS-dependent inflammasome activation during *Salmonella* infection [45]. To test whether this inflammasome activation requires NAIP/NLRC4, we infected WT, *NAIP*^*-/-*^, or *NLRC4*^*-/-*^ THP-1 macrophages with WT *Salmonella* (WT Stm) or *Salmonella* lacking its SPI-1 T3SS (Δ*sipB* Stm) grown under SPI-1-inducing conditions, and assayed for subsequent inflammasome activation (Figs 2, S5 and S6). WT THP-1s infected with WT Stm released high levels of IL-1β, IL-18, and IL-1α and underwent cell death (Figs 2 and S5A–S5D). In *NAIP*^*-/-*^ and *NLRC4*^*-/-*^ THP-1 macrophages infected with WT Stm, we observed a significant decrease but not complete abrogation of secreted IL-1β and IL-18 levels (Fig 2). Corroborating the ELISA data, we observed a decrease but not complete abrogation of cleaved and secreted IL-1β levels in *NAIP*^*-/-*^ THP-1 macrophages infected with WT Stm by western blot analysis (Fig 2E). Levels of IL-1α and cell death were largely unaffected (S5A–S5D Fig). WT, *NAIP*^*-/-*^, and *NLRC4*^*-/-*^ THP-1s released similar levels of the inflammasome-independent cytokine TNF-α (S5E and S5F Fig).

This response was dependent on SPI-1 T3SS translocation into host cells, as cells infected with Δ*sipB* Stm, which lack a component of the translocon, failed to undergo robust inflammasome activation (Figs 2 and S5A–S5D). This may be partly due to reduced uptake of Δ*sipB* Stm into host cells (S6 Fig), given the role of the SPI-1 T3SS in mediating invasion of host cells [53]. Importantly, uptake of either WT Stm or Δ*sipB* Stm across THP-1 genotypes was consistent (S6 Fig), suggesting that the difference in inflammasome activation observed is not due to differential uptake of bacteria between THP-1 genotypes. Overall, these data indicate that NAIP and NLRC4 are partially required for inflammasome responses to *Salmonella* infection in human macrophages, in contrast to what we observe with individual T3SS ligand delivery (Figs 1, S3 and S4), where NAIP/NLRC4 is absolutely required for inflammasome activation. Thus, our data indicate that in addition to the NAIP/NLRC4 inflammasome, *Salmonella* also induces a NAIP/NLRC4-independent inflammasome response, in agreement with published studies [49,50].

### *Salmonella* induces NAIP/NLRC4- and NLRP3-dependent inflammasome activation in human macrophages

**Fig 3:**
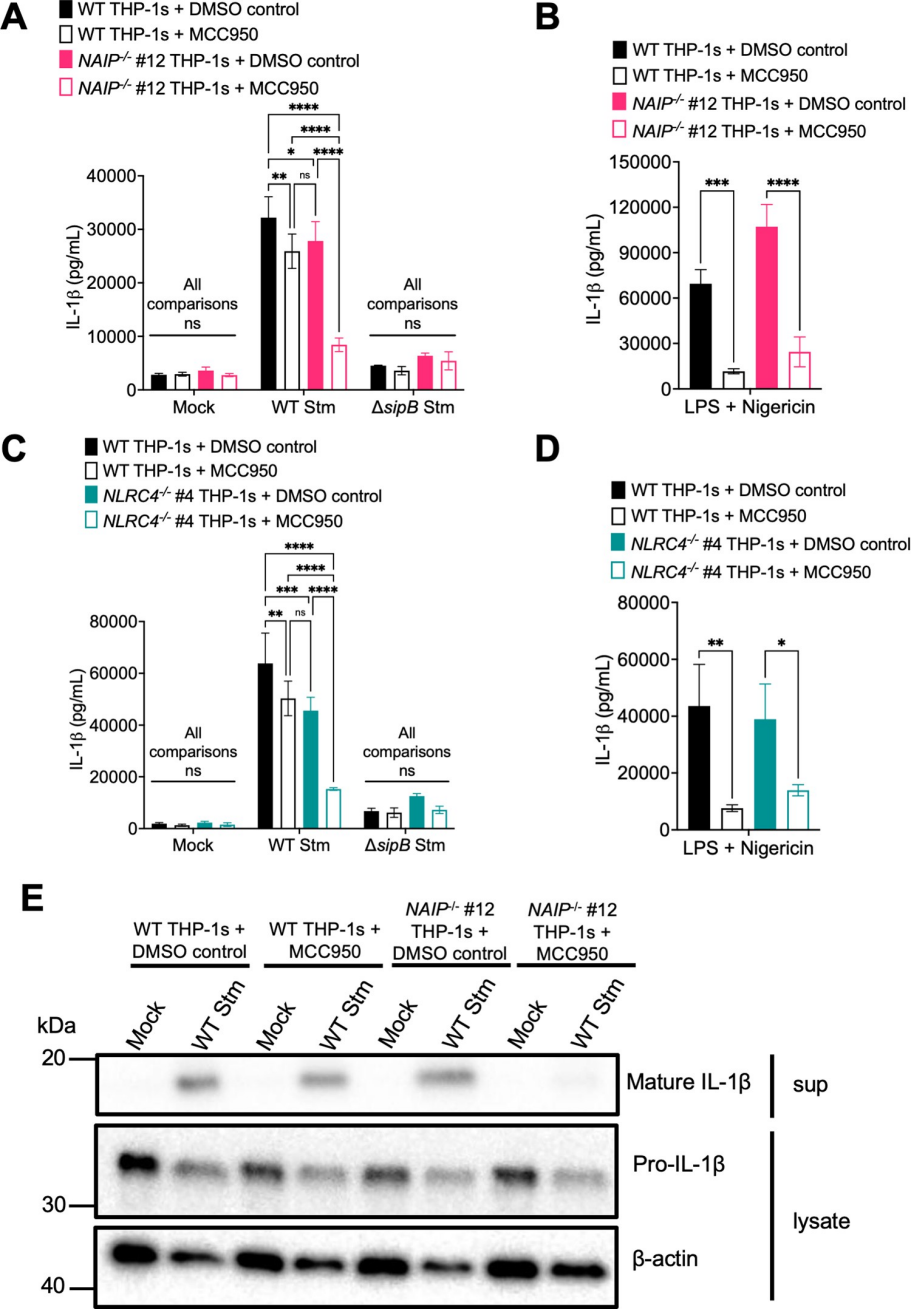
*Salmonella* induces NAIP/NLRC4- and NLRP3-dependent inflammasome activation in human macrophages. (A, C, E) WT, *NAIP*^*-/-*^, or *NLRC4*^*-/-*^ THP-1 monocyte-derived macrophages were primed with 100 ng/mL Pam3CSK4 for 16 hours. One hour prior to infection, cells were treated with 1 μM MCC950, a chemical inhibitor of the NLRP3 inflammasome, or DMSO as a control. Cells were then infected with PBS (Mock), WT *S*. Typhimurium, or Δ*sipB S*. Typhimurium at an MOI = 20 for 6 hours. (B, D) As a control, cells were primed with 500 ng/mL LPS for 4 hours and treated with 10 μM nigericin for 6 hours. (A–D) Release of IL-1β into the supernatant was measured by ELISA. (E) Immunoblot analysis was performed on supernatants (sup) for mature IL-1β and on lysates for pro–IL-1β and β-actin as a loading control. ns–not significant, *p < 0.05, **p < 0.01, ***p < 0.001, ****p < 0.0001 by Tukey’s multiple comparisons test (A, C) or by Šídák’s multiple comparisons test (B, D). Error bars represent the standard deviation of triplicate wells from one experiment. Data shown are representative of at least three independent experiments.

In murine macrophages, *Salmonella* infection activates both the NAIP/NLRC4 and NLRP3 inflammasomes [54,55]. The NAIP/NLRC4 inflammasome is important for early responses to *Salmonella* in the setting of SPI-1 activation, while the NLRP3 inflammasome is important at later timepoints following bacterial replication [54,56]. In human THP-1s, *Salmonella* infection triggers recruitment of both NLRC4 and NLRP3 to the same macromolecular complex [56], and the NAIP and NLRP3 inflammasomes both contribute to inflammasome responses to *Salmonella* [49,50]. The NLRP3 inflammasome can be activated by diverse stimuli during bacterial infection, such as potassium efflux [52,57–60]. To determine if the NAIP/NLRC4-independent inflammasome response we observed in our *Salmonella*-infected human macrophages is NLRP3-dependent, we infected WT, *NAIP*^*-/-*^, or *NLRC4*^*-/-*^ THP-1s with *Salmonella* in the presence of MCC950, a potent chemical inhibitor of the NLRP3 inflammasome [61], or the vehicle control DMSO. We subsequently assayed for inflammasome activation by measuring IL-1α, IL-1β, and IL-18 secretion (Figs 3A, 3C and S7A–S7D). WT THP-1s treated with DMSO control released substantial amounts of IL-1α, IL-1β, and IL-18 when infected with WT Stm. In contrast, infected WT THP-1s treated with MCC950 secreted decreased levels of IL-1α, IL-1β, and IL-18, which are comparable to levels observed in WT Stm-infected *NAIP*^*-/-*^ and *NLRC4*^*-/-*^ THP-1s. (Figs 3A, 3C and S7A–S7D). Interestingly, WT Stm-infected *NAIP*^*-/-*^ and *NLRC4*^*-/-*^ THP-1s treated with MCC950 largely had significantly decreased IL-1α, IL-1β, and IL-18 secretion compared to infected *NAIP*^*-/-*^ or *NLRC4*^*-/-*^ THP-1s treated with DMSO or infected WT THP-1s treated with MCC950 (Figs 3A, 3C, and S7A–S7D). Furthermore, *NAIP*^*-/-*^ and *NLRC4*^*-/-*^ THP-1s treated with MCC950 secreted negligible levels of IL-1α, IL-1β, and IL-18, similar to those observed during Δ*sipB* Stm infection (Figs 3A, 3C and S7A–S7D). WT, *NAIP*^*-/-*^, and *NLRC4*^*-/-*^ THP-1s demonstrated robust IL-1α, IL-1β, and IL-18 secretion in response to LPS + nigericin that was significantly reduced by MCC950 treatment, indicating that this inhibitor effectively blocked NLRP3 inflammasome activation, as expected (Figs 3B, 3D and S7A–S7D). In agreement with our ELISA data, we failed to observe cleaved IL-1β in WT Stm-infected *NAIP*^*-/-*^ THP-1 macrophages treated with MCC950 by western blot analysis (Fig 3E). Release of the inflammasome-independent cytokine TNF-α was similar across the various THP-1 genotypes and treatments following infection (S7E and S7F Fig). Altogether, these data indicate that *Salmonella* infection induces both NAIP/NLRC4- and NLRP3-dependent inflammasome activation in human macrophages, in agreement with previous studies [49,50].

### *Salmonella* induces NAIP/NLRC4- and CASP4/5-dependent inflammasome activation in human macrophages

**Fig 4:**
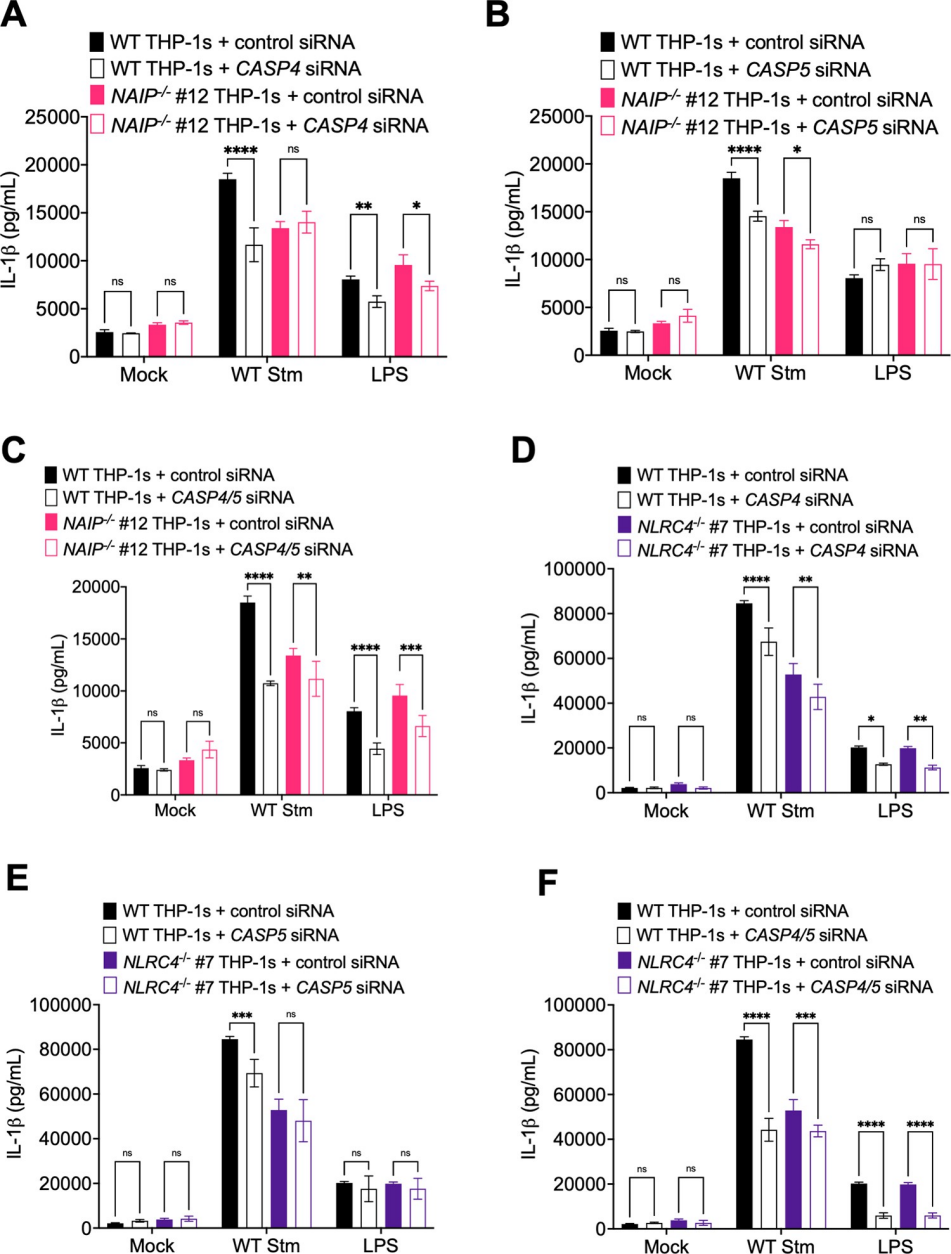
*Salmonella* induces NAIP/NLRC4- and CASP4/5-dependent inflammasome activation in human macrophages. WT, *NAIP*^*-/-*^ (A–C) and *NLRC4*^*-/-*^ (D–F) THP-1 monocyte-derived macrophages were treated with siRNA targeting a control scrambled siRNA, siRNA targeting *CASP4* (A, D) or *CASP5* (B, E), or siRNA targeting both *CASP4* and *CASP5* (C, F) for 48 hours. Cells were primed with 100 ng/mL Pam3CSK4 for 16 hours. Cells were then infected with PBS (Mock) or WT *S*. Typhimurium at an MOI = 20 for 6 hours. Release of IL-1β into the supernatant was measured by ELISA. As a control, cells were transfected with LPS. ns–not significant, *p < 0.05, **p < 0.01, ***p < 0.001, ****p < 0.0001 by Tukey’s multiple comparisons test. Error bars represent the standard deviation of triplicate wells from one experiment. Data shown are representative of at least three independent experiments.

In mice, in addition to the NAIP/NLRC4 and NLRP3 inflammasomes, *Salmonella* infection can also activate the caspase-11 inflammasome [62]. Caspase-11 detects cytosolic LPS and forms the noncanonical inflammasome, which secondarily activates the NLRP3 inflammasome [63–67]. Caspases-4 and 5 are human orthologs of murine caspase-11 [63], and they can also sense cytosolic LPS to form the noncanonical inflammasome and secondarily activate the NLRP3 inflammasome in human cells [64,68,69]. We have previously observed caspase-4-dependent inflammasome activation in response to *Salmonella* infection in primary human macrophages [69], and caspases-4 and 5 also contribute to inflammasome responses to *Salmonella* infection in THP-1s and human intestinal epithelial cells [68,70]. To test the relative contribution of both caspases-4 and 5 to NAIP/NLRC4-independent inflammasome responses during *Salmonella* infection of THP-1 macrophages, we treated WT, *NAIP*^*-/-*^ and *NLRC4*^*-/-*^ THP-1s with siRNAs targeting *CASP4*, *CASP5*, or both, achieving ~70–90% knockdown efficiency at the mRNA level (S8 Fig), and subsequently assayed for IL-1β secretion in response to WT Stm. WT THP-1s treated with either *CASP4* or *CASP5* siRNAs exhibited significantly decreased IL-1β secretion following WT Stm infection relative to WT THP-1s treated with control siRNA (Fig 4A, 4B, 4D and 4E), in agreement with our previous observations in primary human macrophages [69]. *NAIP*^*-/-*^ and *NLRC4*^*-/-*^ THP-1s treated with *CASP5* siRNA showed a slight but significant decrease in IL-1β secretion following *CASP5* siRNA treatment, but not *CASP4* siRNA treatment, compared to control siRNA-treated cells following WT Stm infection (Fig 4A, 4B, 4D and 4E). WT, *NAIP*^*-/-*^, and *NLRC4*^*-/-*^ THP-1s treated with both *CASP4* and *CASP5* siRNAs displayed significantly reduced IL-1β secretion relative to THP-1s treated with a scrambled control siRNA, although inflammasome activation was not completely abrogated when both *CASP4* and *CASP5* were knocked down in *NAIP*^*-/-*^ and *NLRC4*^*-/-*^ THP-1s (Fig 4C and 4F). As a control, we assessed inflammasome activation in response to transfected *E*. *coli* LPS, which activates the caspase-4/5 inflammasome. WT, *NAIP*^*-/-*^, and *NLRC4*^*-/-*^ cells transfected with LPS displayed significantly decreased IL-1β secretion when *CASP4* was silenced, either alone or in conjunction with *CASP5* (Fig 4A, 4C, 4D and 4F), whereas knockdown of *CASP5* alone did not significantly affect IL-1β secretion, as expected [68] (Fig 4B and 4E). Taken together, these data suggest that the caspase-4/5 inflammasome is involved in the NAIP/NLRC4-independent response to *Salmonella*.

### The NAIP/NLRC4 and NLRP3 inflammasomes control *Salmonella* burden within human macrophages

**Fig 5:**
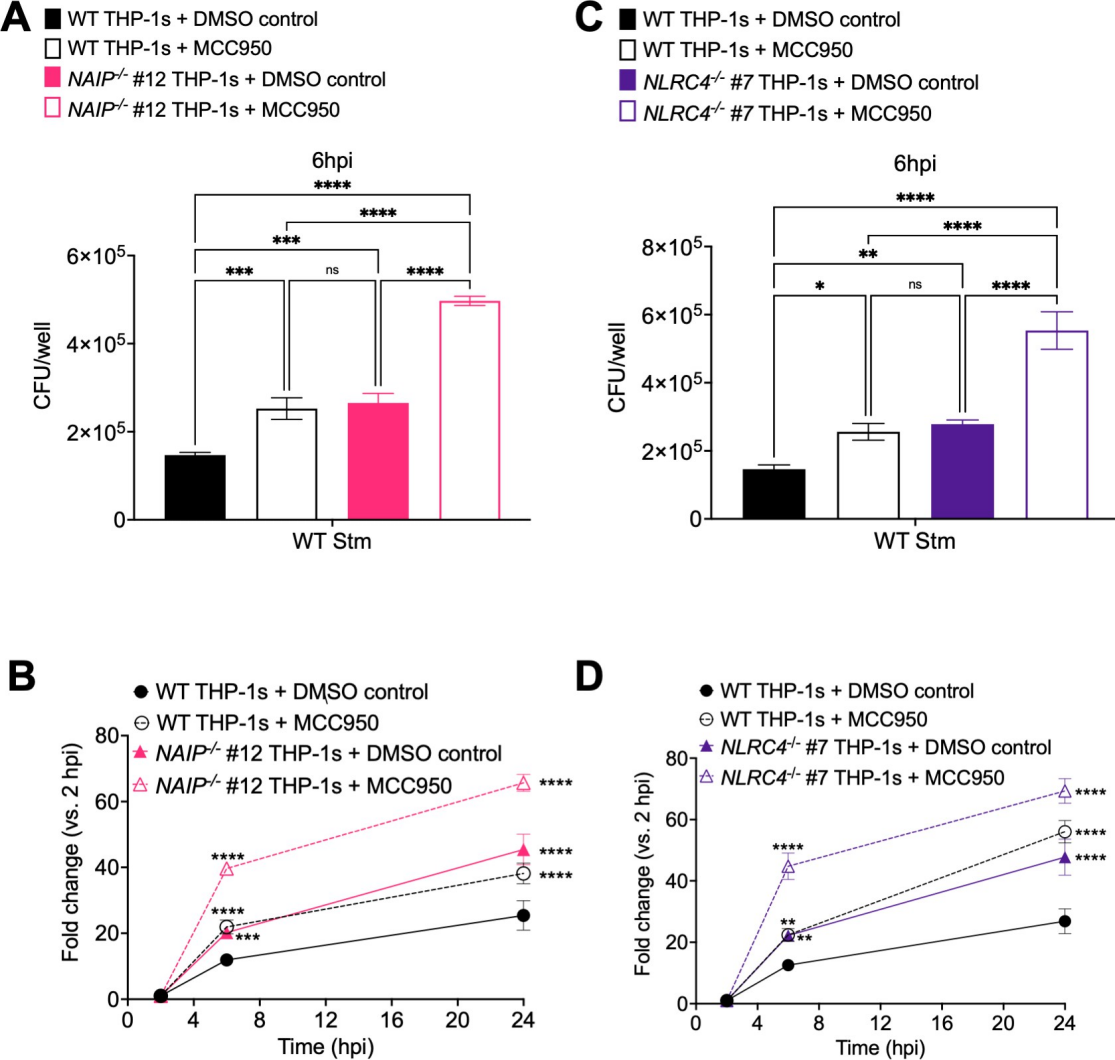
The NAIP/NLRC4 and NLRP3 inflammasomes control *Salmonella* burdens within human macrophages. WT, *NAIP*^*-/-*^ (A, B), and *NLRC4*^*-/-*^(C, D) THP-1 monocyte-derived macrophages were primed with 100 ng/mL Pam3CSK4 for 16 hours. One hour prior to infection, cells were treated with 1 μM MCC950, a chemical inhibitor of the NLRP3 inflammasome, or DMSO as a control. Cells were then infected with PBS (Mock) or WT *S*. Typhimurium at an MOI = 20. Cells were lysed at the indicated time points and bacteria were plated to calculate CFU. (A, C) CFU/well of bacteria at 6 hpi (B, D) Fold change in CFU/well of bacteria at indicated time point, relative to 2 hpi CFU/well. ns–not significant, ***p < 0.001, ****p < 0.0001 by Dunnett’s multiple comparisons test (A, C) or Tukey’s multiple comparisons test (B, D). Error bars represent the standard deviation of triplicate wells from one experiment. Data shown are representative of at least three independent experiments.

One of the mechanisms by which inflammasome activation leads to control of bacterial infection is by restricting intracellular bacterial replication [47,54,71–75]. In mice, the NAIP/NLRC4 inflammasome is important for controlling *Salmonella* replication in the intestine [47,48,72], whereas the NLRP3 inflammasome is dispensable for control of *Salmonella* infection *in vivo* [54,72–74]. Caspases-1 and 11 restrict cytosolic *Salmonella* replication within murine macrophages [76]. Whether inflammasome activation restricts WT *Salmonella* replication in human macrophages is unknown. To test the hypothesis that inflammasome activation restricts *Salmonella* within human macrophages, we infected WT, *NAIP*^*-/-*^, and *NLRC4*^*-/-*^ THP-1 macrophages with WT Stm in the presence or absence of the NLRP3 inhibitor MCC950 and determined the bacterial colony forming units (CFUs) at various timepoints post-infection to assay bacterial burdens. At 2 hours post-infection, we did not observe any differences in bacterial uptake between the different conditions (S9A and S9C Fig). At 6 or 24 hours post-infection, the bacterial burden was the lowest in WT THP-1s, whereas *NAIP*^*-/-*^ and *NLRC4*^*-/-*^ THP-1s harbored significantly higher bacterial burdens (Figs 5A, 5C, S9B and S9D). WT THP-1s treated with MCC950 also contained a significantly higher number of bacterial CFUs, comparable to those in *NAIP*^*-/-*^ and *NLRC4*^*-/-*^ THP-1s (Figs 5A, 5C, S9B and S9D). *NAIP*^*-/-*^ and *NLRC4*^*-/-*^ THP-1s treated with MCC950 had the highest bacterial burdens, which were significantly higher than the bacterial burdens in DMSO control-treated *NAIP*^*-/-*^ and *NLRC4*^*-/-*^ THP-1s or WT THP-s treated with MCC950 (Fig 5A and 5C). We then examined the fold-change in bacterial CFUs at 6 and 24 hours relative to 2 hours post-infection. The fold-change in bacterial CFUs was restricted the most effectively in WT THP-1s, moderately restricted in *NAIP*^*-/-*^ and *NLRC4*^*-/-*^ THP-1s or WT THP-1s treated with MCC950, and the least restricted in *NAIP*^*-/-*^ and *NLRC4*^*-/-*^ THP-1s treated with MCC950 (Fig 5B and 5D).

**Fig 6:**
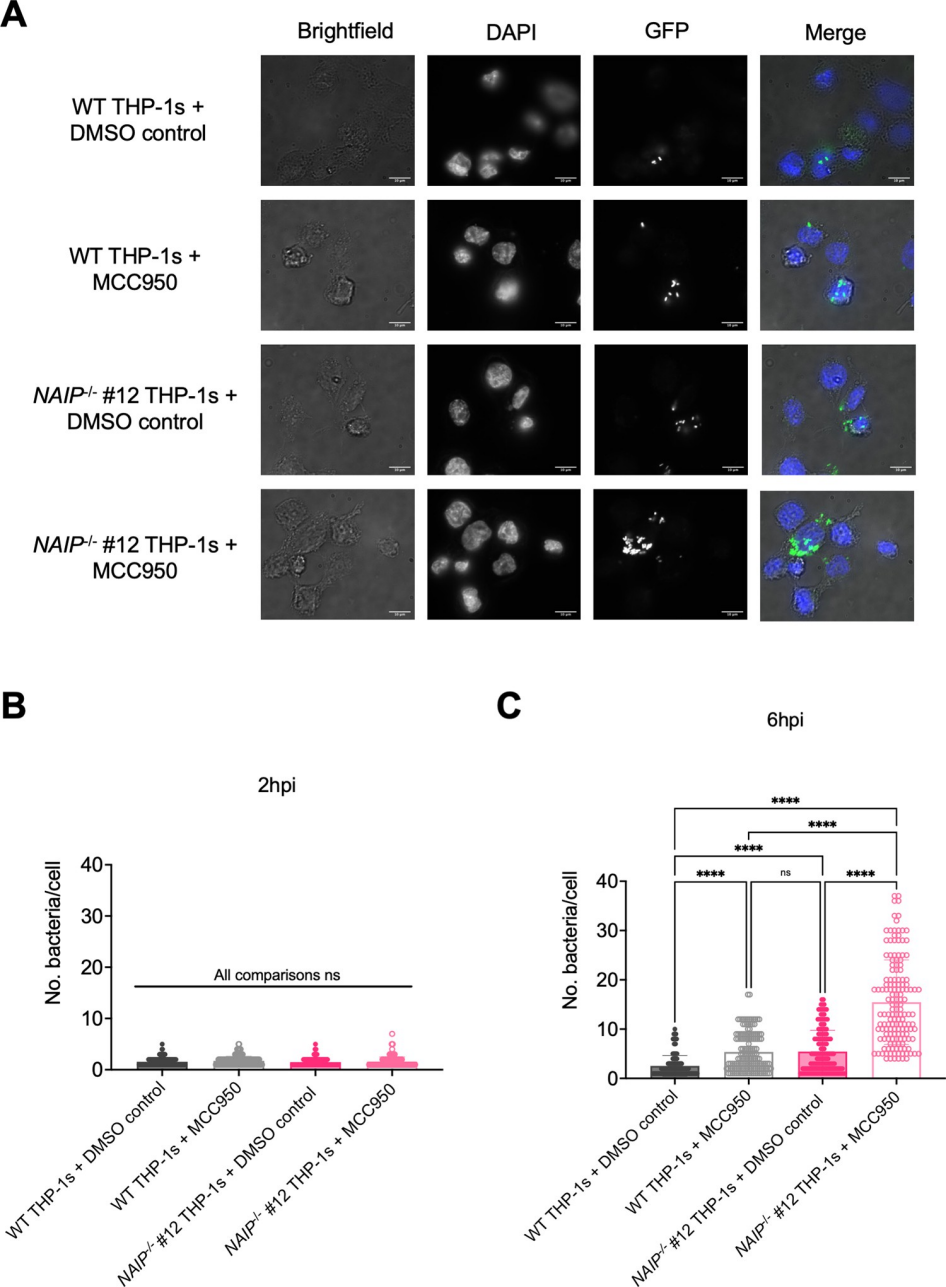
The NAIP/NLRC4 and NLRP3 inflammasomes control *Salmonella* replication within human macrophages. WT and *NAIP*^*-/-*^ THP-1 monocyte-derived macrophages were seeded on glass coverslips and primed with 100 ng/mL Pam3CSK4 for 16 hours. One hour prior to infection, cells were treated with 1 μM MCC950, a chemical inhibitor of the NLRP3 inflammasome, or DMSO as a control. Cells were then infected with PBS (Mock) or WT *S*. Typhimurium expressing GFP at an MOI = 20. Cells were fixed at the indicated time points and stained for DAPI to label DNA (blue). The proportion of infected cells containing GFP-expressing Stm (green) and the number of bacteria per cell were scored by fluorescence microscopy. (A) Representative images from 6hpi are shown. Scale bar represents 10 μm. (B, C) Each small dot represents one infected cell. 150 infected cells were scored for each condition (50 infected cells per coverslip). Bars represent the mean from each condition. (B) Number of bacteria/cell at 2 hpi. (C) Number of bacteria/cell at 6 hpi. ns–not significant, ****p < 0.0001 by Tukey’s multiple comparisons test (B). Data shown are representative of at least three independent experiments.

To further assess bacterial burdens in human macrophages, we infected WT and *NAIP*^*-/-*^ THP-1s with WT Stm expressing GFP in the presence and absence of MCC950 and quantified the number of Stm per cell (Fig 6). Microscopic analysis revealed that DMSO control-treated WT THP-1s contained the lowest number of bacteria per cell, while *NAIP*^*-/-*^ THP-1s treated with MCC950 harbored the highest number of bacteria per cell at 6hpi (Fig 6A and 6C). WT THP-1s treated with MCC950 or DMSO control-treated *NAIP*^*-/-*^ THP-1s contained similar and intermediate levels of bacteria per cell at 6hpi (Fig 6A and 6C). Comparable bacterial burdens were observed across THP-1 genotypes and treatment conditions at 2hpi (Fig 6B). Collectively, these data suggest that both the NAIP/NLRC4 and NLRP3 inflammasomes control intracellular *Salmonella* replication within human macrophages at both early (6 hours post-infection) and late (24 hours post-infection) timepoints.

### *Salmonella* SPI-2 needle protein SsaG activates the NAIP/NLRC4 inflammasome in human macrophages

**Fig 7:**
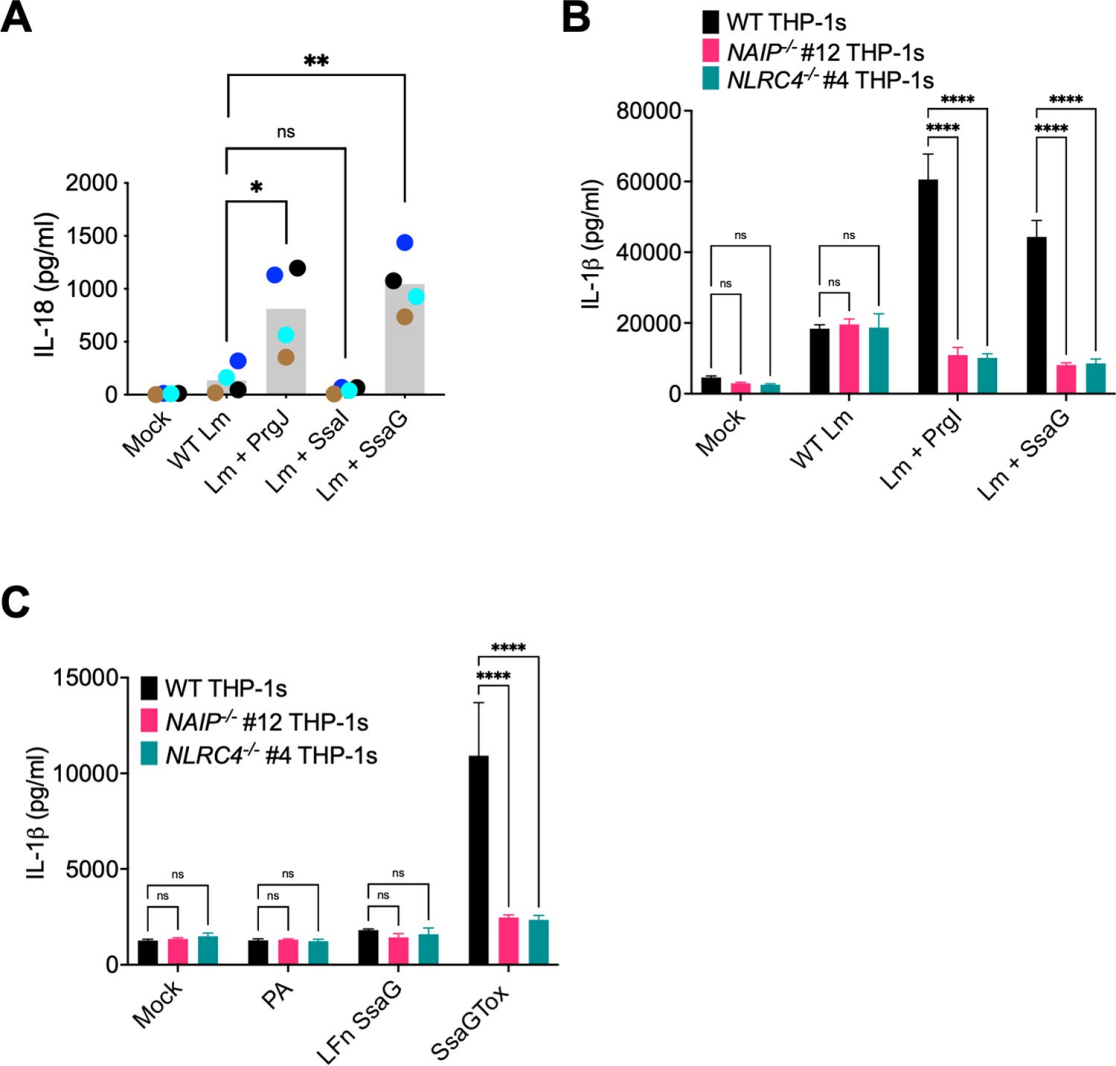
*Salmonella* SPI-2 needle protein SsaG activates the NAIP/NLRC4 inflammasome in human macrophages. (A) Primary hMDMs from four healthy human donors were infected with PBS (Mock), WT *Listeria (*WT Lm*)*, *Listeria* expressing PrgJ (Lm + PrgJ), SsaI (Lm + SsaI), or SsaG (Lm + SsaG) for 16 hours at MOI = 5. Release of IL-18 into the supernatant was measured by ELISA. Each dot represents the mean of individual donors derived from triplicate wells. The grey bar represents the mean of all donors. (B) WT, *NAIP*^*-/-*^, and *NLRC4*^*-/-*^ THP-1 monocyte-derived macrophages were primed with 100 ng/mL Pam3CSK4 for 16 hours. Cells were treated with PBS (Mock), WT *Listeria (*WT Lm*)*, *Listeria* expressing PrgI (Lm + PrgI), or *Listeria* expressing SsaG (Lm + SsaG) for 6 hours at MOI = 20. Release of IL-1β into the supernatant was measured by ELISA. (C) WT, *NAIP*^*-/-*^, and *NLRC4*^*-/-*^ THP-1 monocyte-derived macrophages were primed with 100 ng/mL Pam3CSK4 for 16 hours. Cells were then treated with PBS (Mock), PA alone, LFnSsaG, or PA+LFnSsaG (SsaGTox) for 6 hours. Release of IL-1β into the supernatant was measured by ELISA. ns–not significant, ***p < 0.001, ****p < 0.0001 paired t-test (A) or by Dunnett’s multiple comparisons test (B, C). Data shown are representative of at least three independent experiments.

The *Salmonella* flagellin, SPI-1 T3SS inner rod (PrgJ), and needle (PrgI) proteins all activate NAIP in human macrophages, whereas the *Salmonella* SPI-2 T3SS inner rod protein (SsaI) is not sensed by NAIP [23,27,44–46]. Similarly in mice, SsaI is not sensed by NAIP2 [14]. These findings have led to the model that the SPI-2 T3SS evades inflammasome detection to allow *Salmonella* to replicate or persist in both murine and human macrophages [14,45]. However, our data indicate that the NAIP/NLRC4 inflammasome restricts *Salmonella* replication within human macrophages even at late timepoints, when the SPI-1 T3SS and flagellin are thought to be downregulated [77–85]. As *Salmonella* requires the SPI-2 T3SS to replicate within macrophages [4,9], these data raise the possibility that the NAIP/NLRC4 inflammasome might detect a different SPI-2 T3SS structural component, such as the SPI-2 T3SS needle protein, SsaG. To address whether the human NAIP/NLRC4 inflammasome detects SsaG, we delivered bacterial ligands into the cytosol of primary human monocyte-derived macrophages (hMDMs) derived from anonymous healthy human donors using the Gram-positive bacterium *Listeria monocytogenes*. Following intracellular invasion, *Listeria* (Lm) escapes from its vacuole into the cytosol where it expresses the protein ActA on its surface. Fusing bacterial ligands of interest to the N-terminus of truncated ActA allows these ligands to be delivered into the host cytosol, where they trigger NAIP/NLRC4 inflammasome activation [45,86]. We infected hMDMs with WT control Lm or bacteria expressing PrgJ, SsaI, or SsaG and assayed for inflammasome activation (Figs 7A and S10). hMDMs infected with *Listeria* expressing the SPI-1 T3SS inner rod protein PrgJ induced robust inflammasome activation, indicated by significantly increased IL-18 secretion as well as robust IL-1α and IL-1β secretion compared to mock infection or WT Lm infection alone (Figs 7A and S10), in agreement with our previous findings [45]. In contrast, and as we previously observed [45], *Listeria* expressing the SPI-2 inner rod protein SsaI failed to induce IL-1β, IL-18, and IL-1α secretion or cell death in hMDMs (Figs 7A and S10). Intriguingly, we observed that *Listeria* expressing the SPI-2 needle protein SsaG induced significantly increased IL-18 and robust IL-1α and IL-1β secretion compared to mock infection or WT Lm infection alone (Figs 7A and S10).

To test whether NAIP or NLRC4 are required for inflammasome responses to SsaG, we delivered SsaG into the cytosol of WT, *NAIP*^*-/-*^, and *NLRC4*^*-/-*^ THP-1s using two delivery methods: *Listeria* and the anthrax toxin system (Figs 7B, 7C and S11). We infected WT, *NAIP*^*-/-*^, and *NLRC4*^*-/-*^ THP-1s with WT *Listeria* (Lm) or *Listeria* expressing PrgI or SsaG and assayed for subsequent inflammasome activation by measuring levels of IL-1β, IL-18, and IL-1α secretion and cell death (Figs 7B and S11A–S11C). Infection of WT THP-1s with *Listeria* expressing PrgI or SsaG led to robust release of IL-1 cytokines and cytotoxicity. In contrast, *NAIP*^*-/-*^ and *NLRC4*^*-/-*^ THP-1s infected with *Listeria* expressing PrgI or SsaG released significantly reduced levels of IL-1 cytokines and cell death relative to WT THP-1s that were comparable to the background levels secreted by THP-1s infected with WT Lm (Figs 7B and S11A–S11C). We observed a similar phenotype when we delivered SsaGTox into the cytosol of THP-1s. WT THP-1s released robust levels of IL-1β, IL-18, and IL-1α, whereas *NAIP*^*-/-*^ and *NLRC4*^*-/-*^ THP-1s released negligible levels of IL-1 cytokines (Figs 7C, S11D and S11E). Altogether, these data demonstrate that the SPI-2 needle protein activates the human NAIP/NLRC4 inflammasome, providing evidence that human NAIP can sense and respond to the *Salmonella* SPI-2 T3SS needle.

### NAIP/NLRC4 inflammasome recognition of the SPI-2 T3SS controls intracellular *Salmonella* in human macrophages

**Fig 8:**
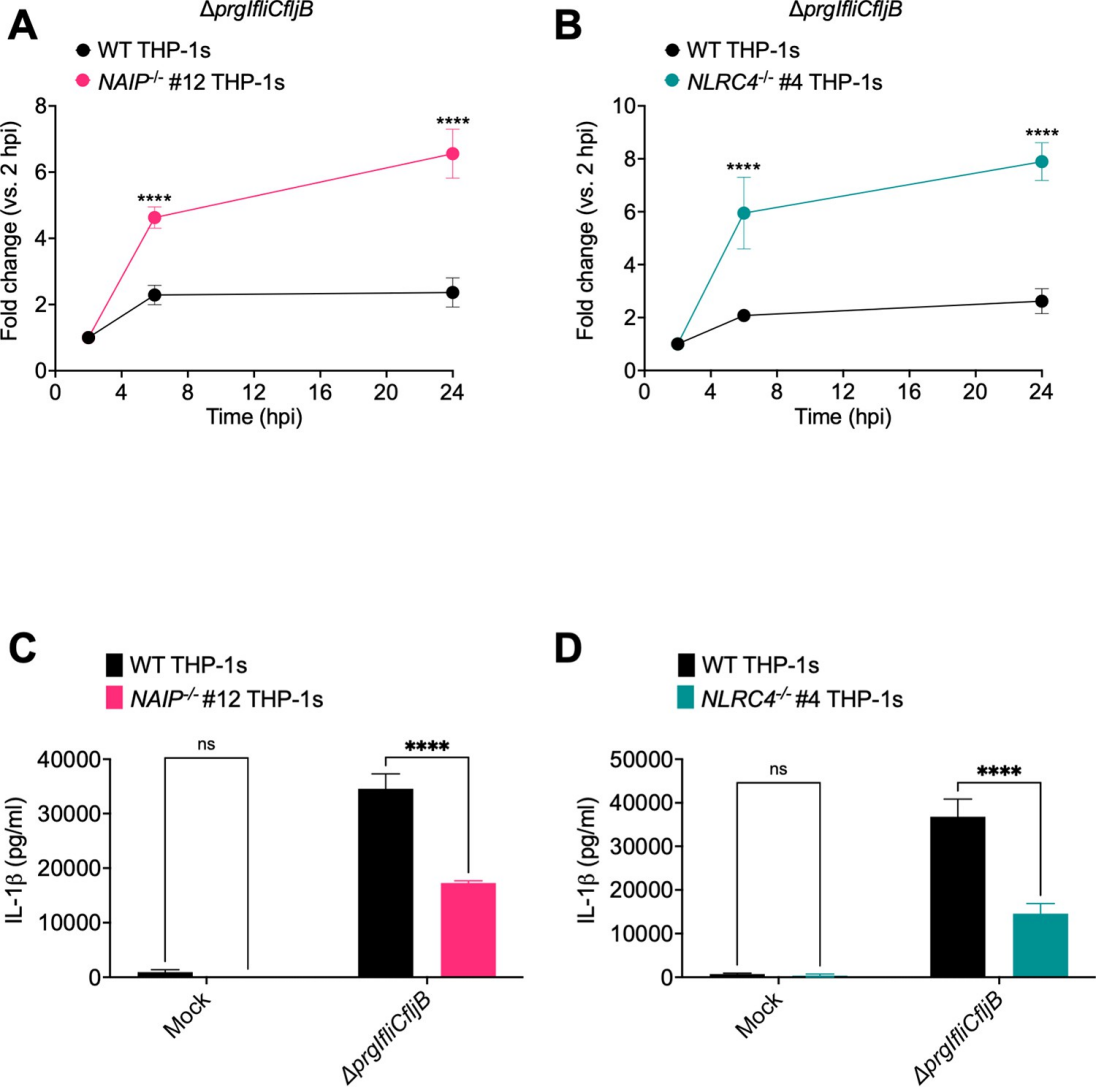
NAIP/NLRC4 inflammasome recognition of the SPI-2 T3SS controls intracellular *Salmonella* in human macrophages. WT, *NAIP*^*-/-*^, and *NLRC4*^*-/-*^ THP-1 monocyte-derived macrophages were primed with 100 ng/mL Pam3CSK4 for 16 hours. Cells were then infected with PBS (Mock) or Δ*prgIfliCfljB S*. Typhimurium at an MOI = 20. (A, B) Cells were lysed at the indicated time points and bacteria were plated to calculate CFU. Fold change in CFU/well of bacteria at indicated time point, relative to 2 hpi CFU/well. (C, D) Release of IL-1β was measured at 24hpi by ELISA. ns–not significant, ****p < 0.0001 by Šídák’s multiple comparisons test.

To determine if NAIP/NLRC4-mediated recognition of the SPI-2 T3SS needle controls intracellular *Salmonella* burdens, we generated a *Salmonella* mutant strain (Δ*prgIfliCfljB*) lacking flagellin and the SPI-1 T3SS needle protein, PrgI. This strain is therefore unable to assemble a functional SPI-1 T3SS, but still expresses a functional SPI-2 T3SS. We infected WT, *NAIP*^*-/-*^, and *NLRC4*^*-/-*^ THP-1 macrophages with Δ*prgIfliCfljB* and determined the CFUs at various timepoints to assay intracellular bacterial burdens (Figs 8A, 8B and S12). The bacterial burden of Δ*prgIfliCfljB* over a 24-hour post-infection time course was controlled the most effectively in WT THP-1s and was significantly less restricted in *NAIP*^*-/-*^ and *NLRC4*^*-/-*^ THP-1s (Figs 8A, 8B and S12). To confirm that inflammasome activation still occurs in response to Δ*prgIfliCfljB*, we infected WT, *NAIP*^*-/-*^, and *NLRC4*^*-/-*^ THP-1 macrophages with Δ*prgIfliCfljB* and measured IL-1β release at 24hpi (Fig 8C and 8D). We observed significant release of IL-1β in WT THP-1s. In contrast, we observed significantly reduced levels of IL-1β release in *NAIP*^*-/-*^ and *NLRC4*^*-/-*^ THP-1 (Fig 8C and 8D). Collectively, our data suggest that there is SPI-1 T3SS/flagellin-independent, NAIP/NLRC4 inflammasome-dependent control of intracellular *Salmonella* burdens in human macrophages, and that NAIP/NLRC4 recognition of the SPI-2 T3SS needle SsaG may mediate such restriction of *Salmonella* within human macrophages.

## Discussion

Our data show that human macrophages engage multiple inflammasome pathways to sense and respond to *Salmonella* infection. Using *NAIP*^*-/-*^ and *NLRC4*^*-/-*^ THP-1s (S1 and S2 Figs), we found inflammasome activation in response to individual T3SS ligands to be entirely dependent on the NAIP/NLRC4 inflammasome in human macrophages (Figs 1, S3 and S4). In contrast, *Salmonella* infection induced activation of inflammasome responses that depended on the collective responses of NAIP/NLRC4, NLRP3, and CASP4/5 (Figs 2–4 and S5–S8). Our findings are consistent with previous studies demonstrating that both NLRC4 and NLRP3 are required for inflammasome responses to *Salmonella* in human macrophages [49,50]. Importantly, our data also reveal that both the NAIP/NLRC4 and NLRP3 inflammasomes contribute to control of *Salmonella* replication in human macrophages (Figs 5, 6 and S9). Furthermore, contrary to the prevailing model that the SPI-2 T3SS as a whole evades NAIP detection, our findings reveal that the NAIP/NLRC4 inflammasome can recognize the *Salmonella* SPI-2 T3SS needle protein SsaG (Figs 7, S10 and S11), and that NAIP/NLRC4-dependent detection of the SPI-2 T3SS controls intracellular *Salmonella* in human macrophages (Figs 8 and S12).

Many Gram-negative bacteria use evolutionarily conserved T3SSs to deliver virulence factors, or effectors, into host cells. We and others have previously shown that T3SS inner rod proteins from various Gram-negative bacteria activate the inflammasome in human macrophages [45,46]. In this study, we used T3SS inner rod, needle, or flagellin proteins from three different Gram-negative bacteria, *Salmonella*, *Burkholderia*, and *Legionella*, and observed that inflammasome activation in response to an isolated ligand is entirely dependent on NAIP/NLRC4 (Figs 1, S3, S4, S7B and S7C). How human NAIP senses and responds to these diverse bacterial structures remains an open question. The *Salmonella* SPI-1 T3SS inner rod (PrgJ), SPI-1 T3SS needle (PrgI), and flagellin proteins exhibit low total sequence conservation, but they all retain several conserved hydrophobic amino acid residues within their structurally homologous C-terminal helices [14, 87–92]. Interestingly, alignment of the amino acid sequences of the SPI-2 T3SS needle protein (SsaG), PrgJ, and PrgI using Clustal Omega revealed that all three proteins contain conserved hydrophobic amino acid residues in their C-terminus (S13A Fig). Specifically, SsaG has C-terminal isoleucine residues like PrgI. These isoleucine residues are important for NAIP-mediated recognition of PrgI [27]. To compare these ligands at the structural level, we examined published three-dimensional structures of PrgJ and PrgI and used PHYRE2 Protein Fold Recognition Server to predict the structure of SsaG. Similar to PrgJ and PrgI, SsaG also displays an alpha-helical structure at its C-terminus (S13B Fig). Unlike these T3SS ligands, the *Salmonella* SPI-2 inner rod protein, SsaI, does not retain such conserved C-terminal residues. Perhaps this is why SsaI is not detected in murine or human macrophages [14,45]. In contrast, our findings indicate that the NAIP/NLRC4 inflammasome detects SsaG. Furthermore, we find that SPI-2-dependent NAIP/NLRC4 inflammasome activation contributes to intracellular control of *Salmonella*.

*Salmonella* infection induces NAIP/NLRC4-, CASP4/5-, and NLRP3-dependent inflammasome activation in human macrophages (Figs 2–4 and S5–S8). This suggests that there is redundancy in the inflammasome pathways when sensing and responding to *Salmonella* infection, such that loss or inhibition of just one inflammasome does not result in severe loss of inflammasome activation in human macrophages. Given our observations with individual ligand delivery (Figs 1, S3 and S4), it is likely that the NAIP/NLRC4 inflammasome is sensing the *Salmonella* T3SS structural components and flagellin. However, it remains unknown how NLRP3 and CASP4/5 inflammasomes are activated in human macrophages during *Salmonella* infection. CASP4/5 detects intracellular LPS [63], but given that *Salmonella* is normally a vacuolar pathogen in macrophages, it is unclear how CASP4/5 may be accessing LPS. In other cell types, *Salmonella* can escape the *Salmonella-*containing vacuole and replicate in the cytosol, leading to CASP4/5 activation [70]. Moreover, other host immune factors, such as guanylate binding proteins (GBPs), can potentiate inflammasome responses to cytoplasmic LPS [65,93–100].

The NLRP3 inflammasome can be activated by a variety of different stimuli, including potassium efflux [52,57–60]. We observed NLRP3 activation induced by *Salmonella* infection that is likely due to activity of the SPI-1 T3SS, since we do not observe inflammasome activation when we infect THP-1 macrophages with Δ*sipB* (Fig 3). Whether the NLRP3 activation is due to the activity of a specific SPI-1 effector or merely due to the collective activity of the SPI-1 effectors in promoting the uptake of *Salmonella* remains unknown. Of note, we observed reduced uptake of Δ*sipB Salmonella* into THP-1 macrophages (S6 Fig). Perhaps, NLRP3 activation is downstream of some *Salmonella*-induced activity that occurs when *Salmonella* successfully and abundantly gets taken up into the cell.

The NLRP3 inflammasome can also be activated downstream of the CASP4/5 inflammasome [64,68]. Given that we observed only partial loss of inflammasome activation in the *NAIP*^*-/-*^ THP-1s treated with siRNA targeting *CASP4* and *CASP5*, we hypothesize that at least part of the NLRP3-dependent response is due to canonical activation (Fig 4), although this partial loss may also be due to incomplete knockdown of *CASP4* and *CASP5*. A recent study, which also found that *Salmonella* infection induces NLRC4- and NLRP3-dependent inflammasome activation in human macrophages, observed that full-length *Salmonella* flagellin can activate the NLRP3 inflammasome [49]. In contrast, we found the response to flagellin to be entirely dependent on the NAIP/NLRC4 inflammasome (Figs 1, S3 and S4). The precise reason for this apparent discrepancy remains to be determined. Notably, in contrast to Gram *et al*. who used full-length flagellin, our study used a truncated flagellin containing only the C-terminal D0 domain, which does not stimulate TLR5 signaling [49,89,101]. It is possible that full-length flagellin used by Gram *et al*., in addition to activating the NAIP/NLRC4 inflammasome, also stimulates TLR5 signaling, perhaps potentiating NLRP3-dependent responses.

We observed NAIP/NLRC4- and NLRP3-dependent restriction of *Salmonella* (Figs 5, 6 and S9), but the mechanism by which inflammasome activation promotes bacterial restriction is unclear. Inflammasome activation often triggers host cell death, thereby eliminating the pathogen’s intracellular replicative niche. *In vivo*, pyroptosis can trigger formation of pore-induced intracellular traps (PITs). These PITs can trap intracellular bacteria that can subsequently be phagocytosed by neutrophils [102]. However, in murine macrophages, inhibition of *Salmonella* replication by caspase-1 and caspase-11 occurs prior to host cell death, indicating that caspase-1 and caspase-11 restrict *Salmonella* through a mechanism distinct from cell death [76]. Another mechanism of inflammasome-dependent restriction may be through promoting phagolysomal maturation. In murine macrophages infected with *Legionella*, NAIP5 activation results in increased colocalization of *Legionella-*containing vacuoles with the lysosomal markers cathepsin-D and Lamp-1 [103,104]. Perhaps a similar process occurs during *Salmonella* infection of human macrophages.

Overall, these data indicate that *Salmonella* infection of human macrophages triggers activation of multiple inflammasomes, and at least two of these inflammasomes, the NAIP/NLRC4, and the NLRP3 inflammasomes, appear to be essential for controlling bacterial replication within macrophages. Furthermore, our data indicate that the human NAIP/NLRC4 inflammasome detects the SPI-2 needle protein SsaG, and that NAIP/NLRC4-mediated detection of the SPI-2 T3SS restricts *Salmonella* within macrophages. Collectively, our findings provide fundamental insight into how *Salmonella* is sensed and restricted by human macrophages. Moreover, these results offer a foundation for further understanding of how each of these pathways is activated and how these inflammasomes interact to mediate downstream responses that promote control of *Salmonella* infection in human macrophages.

## Materials and methods

### Ethics statement

All studies involving primary human monocyte-derived macrophages (hMDMs) were performed in compliance with the requirements of the US Department of Health and Human Services and the principles expressed in the Declaration of Helsinki. hMDMs were derived from samples obtained from the University of Pennsylvania Human Immunology Core. These samples are considered to be a secondary use of deidentified human specimens and are exempt via Title 55 Part 46, Subpart A of 46.101 (b) of the Code of Federal Regulations.

### Bacterial strains and growth conditions

Targeted deletion strains used in this study were made on the *Salmonella enterica* serovar Typhimurium SL1344 strain background. The Δ*prgIfliCfljB* strain was engineered using the Δ*fliCfljB* background [105], in which the SPI-1 T3SS needle, *prgI*, was deleted through a chloramphenicol resistance cassette insertion into *prgI* (*fliCfljBprgI*::CmR) using standard methods [106].

WT, Δ*sipB* [107], and Δ*prgIfliCfljB* isogenic strains were routinely grown overnight in Luria-Bertani (LB) broth with streptomycin (100 μg/ml) at 37°C. For infection of cultured cells, overnight cultures were diluted in LB containing 300 mM NaCl and grown standing for 3 hours at 37°C to induce SPI-1 expression [108].

*Listeria monocytogenes* WT and isogenic strains on the 10403S background were cultured in brain heart infusion (BHI) medium [86]. The *Listeria* strain encoding the heterologous bacterial ligand *S*. Typhimurium PrgJ translationally fused to the truncated N-terminus of ActA and under the control of the *actA* promoter was used [86]. The *Listeria* strains expressing *S*. Typhimurium SsaI and SsaG were constructed using codon-optimized gene fragments (IDT) cloned into the pPL2 vector and introduced into *Listeria* as previously described [86,109].

### Cell culture of THP-1s

THP-1s (TIB-202; American Type Culture Collection) were maintained in RPMI supplemented with 10% (vol/vol) heat-inactivated FBS, 0.05 nM β-mercaptoethanol, 100 IU/mL penicillin, and 100 μg/mL streptomycin at 37°C in a humidified incubator. Two days before experimentation, the cells were replated in media without antibiotics in a 48-well plate at a concentration of 2 × 10^5^ cells/well and incubated with phorbol 12-myristate 13-acetate (PMA) for 24 hours to allow differentiation into macrophages. Macrophages were primed with 100 ng/mL Pam3CSK4 (Invivogen) for 16 hours prior to bacterial infections or anthrax toxin treatments. For experiments involving LPS, cells were pretreated with 500 ng/mL LPS (Sigma-Aldrich) for 3 hours. For experiments involving Nigericin, cells were treated with 10 μM Nigericin (EMD Millipore) for 6 hours. For experiments involving MCC950, cells were treated with 1 μM MCC950 (Sigma Aldrich) 1 hour prior to infection.

### Cell culture of primary human monocyte-derived macrophages (hMDMs)

Purified human monocytes from de-identified healthy human donors were obtained from the University of Pennsylvania Human Immunology Core. Monocytes were cultured in RPMI supplemented with 10% (vol/vol) heat-inactivated FBS, 2 mM L-glutamine, 100 IU/mL penicillin, 100 μg/ml streptomycin, and 50 ng/ml recombinant human M-CSF (Gemini Bio-Products) for 6 days to promote differentiation into hMDMs. One day prior to infection, adherent hMDMs were replated in media with 25 ng/ml human M-CSF lacking antibiotics at 1.0 × 10^5^ cells/well in a 48-well plate.

### Bacterial infections

Overnight cultures of *Salmonella* were diluted into LB broth containing 300 mM NaCl and grown for 3 hours standing at 37°C to induce SPI-1 expression [108]. Overnight cultures of *L*. *monocytogenes* were diluted and grown for 3 hours in BHI. All cultures were pelleted at 6,010 × *g* for 3 minutes, washed once with PBS, and resuspended in PBS. THP-1s were infected with *S*. Typhimurium or *L*. *monocytogenes* at a multiplicity of infection (MOI) of 20. hMDMs were infected with *L*. *monocytogenes* at an MOI of 5. Infected cells were centrifuged at 290 × *g* for 10 min and incubated at 37°C. 1 hour post-infection, cells were treated with 100 ng/mL or 50 ng/mL of gentamicin to kill any extracellular *S*. Typhimurium or *L*. *monocytogenes* respectively. *Salmonella* and *Listeria* infections in THP-1s proceeded at 37°C for 6 hours. *Listeria* infection of hMDMs proceeded at 37°C for 16 hours. For all experiments, control cells were mock-infected with PBS.

### LFn-SsaG construct design and cloning

A construct encoding 6xHis-LFn-SsaG-6xHis was generated by sequential, ligation-free cloning. Briefly, the N-terminus of anthrax lethal factor (LFn) was amplified from FBDual-LFn-PrgJ and cloned into a linearized pOPIN-B *E*. *coli* expression vector [28,110]. Full-length SsaG was cloned into pOPIN-B-LFn from *Salmonella enterica* serovar Typhimurium SL1344 whole genomic DNA with addition of a C-terminal 6xHis tag.

### Purification of His-tagged LFn-SsaG

Recombinant 6xHis-LFn-SsaG-6xHis was obtained by transforming pOPINB-LFn-SsaG into Rosetta DE3 competent *E*. *coli*. Cells were grown to an OD_600_ of 0.7, at which point they were induced with 0.2 mM isopropyl β-d-1-thiogalactopyranoside (IPTG) and grown overnight for 18 hours at 18°C shaking at 180 rpm. Cells were pelleted and resuspended in a buffer solution containing 25 mM Tris (pH 8 at 4°C), 200 mM sodium chloride, and 2 mM β-Mercaptoethanol (BME) and lysed by sonication. Crude lysate was allowed to bind to HisPur NI-NTA resin at 4°C for 30 minutes and washed with a buffer solution containing 25 mM Tris (pH 8 at 4°C), 400 mM sodium chloride, and 2 mM BME.

A step gradient elution was adapted from a Cold Spring Harbor protocol [111]. Briefly, 2 ml each of 25 mM Tris (pH 8 at 4°C), 200 mM sodium chloride, 2 mM BME buffer with either 50 mM, 100 mM, 150 mM, 200 mM, 250 mM, 300 mM, or 500 mM imidazole were incubated sequentially on the resin for 3 minutes, and eluted into individual fractions. SDS-PAGE gel determined that the cleanest fractions were 200 mM and 250 mM imidazole. The selected fractions were dialyzed in 25 mM sodium phosphate buffer (pH 7.4) containing 0.5 mM EDTA over-night, concentrated by centrifugation, and flash frozen. Protein concentration was determined by NanoDrop.

### Anthrax toxin-mediated delivery of bacterial ligands

Recombinant proteins (PA, LFn-FlaA^310-475^, LFn-PrgJ, and LFn-YscF) were kindly provided by Russell Vance [28]. LFn-SsaG was synthesized as described above. PA and LFn doses for *in vitro* delivery were: 1 μg/mL PA for FlaTox; 4 μg/mL PA for PrgJTox, YscFTox, and SsaGTox; 500 ng/mL LFn-FlaA^310-475^; 8 ng/mL LFn-PrgJ; 200 ng/mL LFn-YscF; 1.36 μg/mL LFn-SsaG.

### siRNA-mediated knockdown of genes

All Silencer Select siRNA oligos were purchased from Ambion (Life Technologies). For *CASP4*, siRNA ID# s2412 was used. For *CASP5*, siRNA ID# s2417 was used. The two Silencer Select negative control siRNAs (Silencer Select Negative Control No. 1 siRNA and Silencer Select Negative Control No. 2 siRNA) were used as a control. Two days before infection, 30 nM of siRNA was transfected into macrophages using Lipofectamine RNAiMAX transfection reagent (Thermo Fisher Scientific) following the manufacturer’s protocol. 16 hours before infection, the media was replaced with fresh antibiotic-free media containing 100 ng/ml Pam3CSK4. In parallel, siRNA-transfected cells were also transfected with 2 μg/ml of *E*. *coli* LPS strain W3110 (kindly provided by Robert Ernst) using FuGENE HD transfection reagent (Promega) for 6 hours.

### Bacterial intracellular burden assay

Cells were infected with WT or Δ*prgIfliCfljB S*. Typhimurium as usual at an MOI of 20.1 hour post-infection, cells were treated with 100 μg/ml of gentamicin to kill any extracellular bacteria. 2 hours post-infection, the media was replaced with fresh media containing 10 μg/ml of gentamicin. At the indicated time points, cells were lysed with PBS containing 0.5% Triton to collect all intracellular bacteria. Harvested bacteria were serially diluted in PBS and plated on LB agar plates containing streptomycin (100 μg/ml) to enumerate colony forming units (CFUs). Plates were incubated at 37°C overnight and then CFUs were counted.

### ELISAs

Harvested supernatants from infected cells were assayed using ELISA kits for human IL-1α (R&D Systems), IL-18 (R&D Systems), IL-1β (BD Biosciences), and TNF-α (R&D Systems).

### LDH cytotoxicity assays

Harvested supernatants from infected cells were assayed for cytotoxicity by measuring loss of cellular membrane integrity via lactate dehydrogenase (LDH) activity. LDH release was quantified using an LDH Cytotoxicity Detection Kit (Clontech) according to the manufacturer’s instructions and normalized to mock-infected cells.

### Quantitative RT-PCR analysis

RNA was isolated using the RNeasy Plus Mini Kit (Qiagen) following the manufacturer’s instructions. Cells were lysed in 350 μL RLT buffer with β-mercaptoethanol and centrifuged through a QIAshredder spin column (Qiagen). cDNA was synthesized from isolated RNA using SuperScript II Reverse Transcriptase (Invitrogen) following the manufacturer’s protocol. Quantitative PCR was conducted with the CFX96 real-time system from Bio-Rad using the SsoFast EvaGreen Supermix with Low ROX (Bio-Rad). For analysis, mRNA levels of siRNA-treated cells were normalized to housekeeping gene *HPRT* and control siRNA-treated cells using the 2^−ΔΔCT^ (cycle threshold) method [112] to calculate knockdown efficiency. The following primers from PrimerBank were used. The PrimerBank identifications are *CASP4* (73622124c1), and *CASP5* (209870072c2), and *HPRT* (164518913c1); all 5′–3′:

*CASP4* forward: CAAGAGAAGCAACGTATGGCA

*CASP4* reverse: AGGCAGATGGTCAAACTCTGTA

*CASP5* forward: TTCAACACCACATAACGTGTCC

*CASP5* reverse: GTCAAGGTTGCTCGTTCTATGG

*HPRT* forward: CCTGGCGTCGTGATTAGTGAT

*HPRT* reverse: AGACGTTCAGTCCTGTCCATAA

### Immunoblot analysis

Cell lysates were harvested for immunoblot analysis by adding 1X SDS/PAGE sample buffer to cells following infection. Cells were incubated and infected in serum-free media to collect supernatant samples. Supernatant samples were centrifuged at 200 × *g* to pellet any cell debris. The supernatant was then treated with trichloroacetic acid (TCA) (25 μL of TCA for 300 μL of supernatant) overnight at 4°C. The next day, the samples were centrifuged at maximum speed (15871 × *g*) for 15 minutes at 4°C. Precipitated supernatant pellets were washed with ice-cold acetone, centrifuged at maximum speed (15871 × *g*) for 10 minutes at 4°C, and resuspended in 1X SDS/PAGE sample buffer. All protein samples (lysates and supernatants) were boiled for 5 minutes. Samples were separated by SDS/PAGE on a 12% (vol/vol) acrylamide gel, and transferred to PVDF Immobilon-P membranes (Millipore). Primary antibodies specific for human IL-1β (#8516; R&D Systems) and β-actin (4967L; Cell Signaling) and HRP-conjugated secondary antibodies anti-mouse IgG (F00011; Cell Signaling) and anti-rabbit IgG (7074S; Cell Signaling) were used. ECL Western Blotting Substrate (Pierce Thermo Scientific) was used as the HRP substrate for detection.

### Fluorescent microscopy of intracellular *Salmonella*

Two days prior to infection, 3 × 10^5^ cells/well plated on glass coverslips in a 24-well plate. Cells were treated with PMA and primed with 100 ng/mL Pam3CSK4 as described above. Cells were infected with WT *Salmonella* pFPV25.1 (*Salmonella* constitutively expressing GFP) at an MOI of 20 as described above. At the indicated timepoints following infection, cells were washed 2 times with PBS and fixed with 4% paraformaldehyde for 10 min at 37°C. Following fixation, cells were mounted on glass slides with DAPI mounting medium (Sigma Fluoroshield). Coverslips were imaged on an inverted fluorescence microscope (IX81; Olympus) and images were collected using a high-resolution charge-coupled-device camera (FAST1394; QImaging) at a magnification of 60×. All images were analyzed and presented using SlideBook (version 5.0) software (Intelligent Imaging Innovations, Inc.) and scale bars were added using ImageJ software. The proportion of infected cells containing GFP-expressing Stm (green) were scored by counting 50 infected cells per coverslip. 150 total infected cells were scored for each condition.

### Statistical analysis

Prism 9.2.0 (GraphPad Software) was utilized for the graphing of data and all statistical analyses. Statistical significance for experiments with THP-1s and hMDMs was determined using the appropriate test and are indicated in each figure legend. Differences were considered statistically significant if the *p* value was <0.05.

## Supporting information

S1 Fig Validation of *NAIP* mutant THP-1 single cell clones generated with CRISPR/Cas9 genome editing. (A) Schematic representation of the *NAIP* gene with exons (filled boxes) and introns (filled lines). gRNA target sequence is highlighted in red. (B) Sequence alignments of WT THP-1s and *NAIP*^*-/-*^ clone #12 are shown for both alleles. Red boxes represent the mutated region. Purple text represents the predicted impact of the mutation on the amino acid sequence. (C) qRT-PCR was performed to quantitate *NAIP* mRNA levels in WT THP-1s and *NAIP*^*-/-*^ THP-1s. For the *NAIP*^*-/-*^ THP-1s, *NAIP* mRNA levels were normalized to human HPRT mRNA levels and WT THP-1s.
(TIF)

S2 Fig Validation of *NLRC4* mutant THP-1 single cell clones generated with CRISPR/Cas9-mediated genome editing. (A) Schematic representation of the *NLRC4* gene with exons (filled boxes) and introns (lines). gRNA target sequence is highlighted in red. (B-C) Sequence alignments of WT THP-1s and *NLRC4*^*-/-*^ clones are shown for both alleles per clone. Red boxes highlight the mutated region. Purple text represents the predicted impact of the mutation on the amino acid sequence. (D) Immunoblot analysis was performed on cell lysates for human NLRC4, and β-actin as a loading control.
(TIF)

S3 Fig (related to Fig 1) NAIP is necessary for inflammasome responses to T3SS ligands in human macrophages. WT or *NAIP*^*-/-*^ THP-1 monocyte-derived macrophages were primed with 100 ng/ml Pam3CSK4 for 16 hours. Cells were then treated with PBS (Mock), PA alone, LFn FlaA^310–475^ (LFn FlaA) alone, LFn PrgJ alone, LF YscF alone, PA+LFn FlaA^310–475^ (FlaTox), PA+LFn PrgJ (PrgJTox), or PA+LFn YscF (YscFTox) for 6 hours. (A, B, D) Release of cytokines IL-18, IL-1α, and TNF-α into the supernatant were measured by ELISA. (C) Cell death (percentage cytotoxicity) was measured by lactate dehydrogenase release assay and normalized to Mock-treated cells. ns–not significant, **p < 0.01, ****p < 0.0001 by Šídák’s multiple comparisons test. Data shown are representative of at least three independent experiments.
(TIF)

S4 Fig (related to Fig 1) NLRC4 is necessary for inflammasome responses to T3SS ligands in human macrophages. WT or two independent clones of *NLRC4*^*-/-*^ THP-1 monocyte-derived macrophages were primed with 100 ng/ml Pam3CSK4 for 16 hours. Cells were then treated with PBS (Mock), PA alone, LFn FlaA^310–475^ alone, LFn PrgJ alone, LFn YscF alone, PA+LFn FlaA^310–475^ (FlaTox), PA+LFn PrgJ (PrgJTox), or PA+LFn YscF (YscFTox) for 6 hours. (A, B, D) Release of cytokines IL-18, IL-1α, and TNF-α into the supernatant were measured by ELISA. (C) Cell death (percentage cytotoxicity) was measured by lactate dehydrogenase release assay and normalized to Mock-treated cells. ns–not significant, *p < 0.05, **p < 0.01, ***p < 0.001, ****p < 0.0001 by Dunnett’s multiple comparisons test (A-C). Data shown are representative of at least three independent experiments.
(TIF)

S5 Fig (related to Fig 2) NAIP and NLRC4 are partially required for inflammasome activation during *Salmonella* infection in human macrophages. WT, *NAIP*^*-/-*^, or two independent clones of *NLRC4*^*-/-*^ THP-1 monocyte-derived macrophages were primed with 100 ng/mL Pam3CSK4 for 16 hours. Cells were then infected with PBS (Mock), WT *S*. Typhimurium, or Δ*sipB S*. Typhimurium at an MOI = 20 for 6 hours. As a control, cells were primed with 500 ng/mL LPS for 4 hours and treated with 10 μM nigericin for 6 hours. (A, C, E, F) Release of cytokines IL-1α and TNF-α into the supernatant were measured by ELISA. (B, D) Cell death (percentage cytotoxity) was measured by lactate dehydrogenase release assay and normalized to Mock-treated cells. ns–not significant, *p < 0.05, ***p < 0.001 by Šídák’s multiple comparisons test (A, B, E) or by Dunnett’s multiple comparisons test (C, D, F). Data shown are representative of at least three independent experiments.
(TIF)

S6 Fig (related to Fig 2). Uptake of *Salmonella* into THP-1 macrophages. WT, *NAIP*^*-/-*^, and two independent clones of *NLRC4*^*-/-*^ THP-1 monocyte-derived macrophages were primed with 100 ng/mL Pam3CSK4 for 16 hours. Cells were then infected with WT *S*. Typhimurium or Δ*sipB S*. Typhimurium at an MOI = 20. Cells were lysed at the 2 hours post-infection and bacteria were plated to calculate CFU. ns–not significant, *p < 0.05, ***p < 0.001 by Tukey’s multiple comparisons test. Data shown are representative of at least three independent experiments.
(TIF)

S7 Fig (related to Fig 3) *Salmonella* induces NAIP/NLRC4- and NLRP3-dependent inflammasome activation in human macrophages. WT, *NAIP*^*-/-*^, or *NLRC4*^*-/-*^ THP-1 monocyte-derived macrophages were primed with 100 ng/mL Pam3CSK4 for 16 hours. One hour prior to infection, cells were treated with 1 μM MCC950, a chemical inhibitor of the NLRP3 inflammasome. Cells were then infected with PBS (Mock), WT *S*. Typhimurium, or Δ*sipB S*. Typhimurium at an MOI = 20 for 6 hours. (B) As a control, cells were primed with 500 ng/mL LPS for 4 hours and treated with 10 μM nigericin for 6 hours. (A-F) Release of cytokines IL-18, IL-1α, and TNF-α into the supernatant were measured by ELISA. ns–not significant, *p < 0.05, **p < 0.01, ***p < 0.001, ****p < 0.0001 by Tukey’s multiple comparisons test.
(TIF)

S8 Fig (related to Fig 4) Knockdown efficiencies of siRNA-mediated silencing of *CASP4* and *CASP5* in human macrophages. Knockdown efficiencies following siRNA treatment were measured by qRT-PCR and normalized to housekeeping gene *HPRT*, and calculated relative to control-siRNA-treated cells. (A) siRNA targeting *CASP4* or *CASP5* in WT vs *NAIP*^*-/-*^ #12. (B) siRNA targeting *CASP4* and *CASP5* in WT vs *NAIP*^*-/-*^ #12. (C) siRNA targeting *CASP4* or *CASP5* in WT vs *NLRC4*^*-/-*^ #7. (D) siRNA targeting *CASP4* and *CASP5* in WT vs *NLRC4*^*-/-*^ #7. Data shown are averages of at least three independent experiments.
(TIF)

S9 Fig (related to Fig 5) NAIP and NLRP3 restrict replication of *Salmonella* in human macrophages. WT, *NAIP*^*-/-*^ (A, B), and *NLRC4*^*-/-*^ #7 (C, D) THP-1 monocyte-derived macrophages were primed with 100 ng/ml Pam3CSK4 for 16 hours. One hour prior to infection, cells were treated with 1 μM MCC950 or DMSO as a control. Cells were then infected with WT *S*. Typhimurium at an MOI = 20. Cells were lysed at the indicated time points and bacterial were plated to calculate CFU. (A, C) CFU/well of bacteria at 2 hpi (B, D) CFU/well of bacteria at 24 hpi. *p < 0.05, **p < 0.01, ***p < 0.001, ****p < 0.0001 by Tukey’s multiple comparisons test. Data shown are representative of at least three independent experiments.
(TIF)

S10 Fig (related to Fig 7A) *Salmonella* SPI-2 needle protein SsaG activates the inflammasome in human macrophages. Primary hMDMs from four healthy human donors was infected with PBS (Mock), WT *Listeria* (WT Lm), *Listeria* expressing PrgJ (Lm + PrgJ), SsaI (Lm + SsaI), or SsaG (Lm + SsaG) for 16 hours at MOI = 5. Each dot represents the triplicate mean of one donor. The grey bar represents the mean of all donors. Release of cytokines IL-1β and IL-1α was measured by ELISA. *p* values based on paired t-tests.
(TIF)

S11 Fig (related to Fig 7B and 7C) NAIP/NLRC4 are necessary for inflammasome responses to the *Salmonella* SPI-2 needle protein SsaG in human macrophages. WT, *NAIP*^*-/-*^, or *NLRC4*^*-/-*^ THP-1 monocyte-derived macrophages were primed with 100 ng/ml Pam3CSK4 for 16 hours. (A–C) Cells were then treated with PBS (Mock), WT *Listeria* (WT Lm), *Listeria* expressing PrgI (Lm + PrgJ) or SsaG (Lm + SsaG) for 6 hours at MOI = 20. (A, B) Release of cytokines IL-18 and IL-1α was measured by ELISA. (C) Cell death was measured by lactate dehydrogenase (LDH) release. (D, E) Cells were treated with PBS (Mock), PA alone, LFn SsaG alone, PA+LFn SsaG (SsaGTox) for 6 hours. Release of cytokines IL-18 and IL-1α was measured by ELISA. ns–not significant, *p < 0.05, **p < 0.01, ***p < 0.001, ****p < 0.0001 by Dunnett’s multiple comparisons test. Data shown are representative of at least three independent experiments.
(TIF)

S12 Fig (related to Fig 8) NAIP/NLRC4 inflammasome recognition of the SPI-2 T3SS restricts *Salmonella* replication in human macrophages. WT, *NAIP*^*-/-*^ (A–C) and *NLRC4*^*-/-*^ (D–F) THP-1 monocyte-derived macrophages were primed with 100 ng/ml Pam3CSK4 for 16 hours. Cells were then infected with a SPI-1 T3SS/flagellin-deficient strain of *S*. Typhimurium, Δ*prgIfliCfljB* at an MOI = 20. (A, D) CFU/well of bacteria at 2 hpi (B, E) CFU/well of bacteria at 6 hpi. (C, F) CFU/well of bacteria at 24 hpi. **p < 0.01, ***p < 0.001, by unpaired t-test. Data shown are representative of at least three independent experiments.
(TIF)

S13 Fig Sequence alignment and three-dimensional structural prediction of SsaG. (A) The primary sequences of PrgJ, PrgI, and SsaG were aligned using Multiple Sequence Alignment by Clustal Omega. * indicates single, *fully conserved* residue,: indicates conservation between groups of *strongly* similar properties, and. indicates conservation between groups of *weakly* similar properties. Small, hydrophobic residues are indicated in red (AVFPMILW). Acidic residues are indicated in blue (DE). Basic residues are indicated in magenta (RK). The remaining residues are indicated in green (STYHCNGQ). (B) The three-dimensional structure of SsaG was predicted with high confidence and high coverage using the PHYRE2 server. The structure is colored from N to C terminus using the colors of the rainbow (red, orange, yellow, green, and blue).
(TIF)

S1 File Supplemental Materials and Methods. (DOCX)
