# Supplementary figures and images for: Human NAIP/NLRC4 and NLRP3 inflammasomes detect Salmonella type III secretion system activities to restrict intracellular bacterial replication
Source: PLoS Pathog. 2022 Jan 24;18(1):e1009718. doi: 10.1371/journal.ppat.1009718 (PMC8812861; doi:10.1371/journal.ppat.1009718)

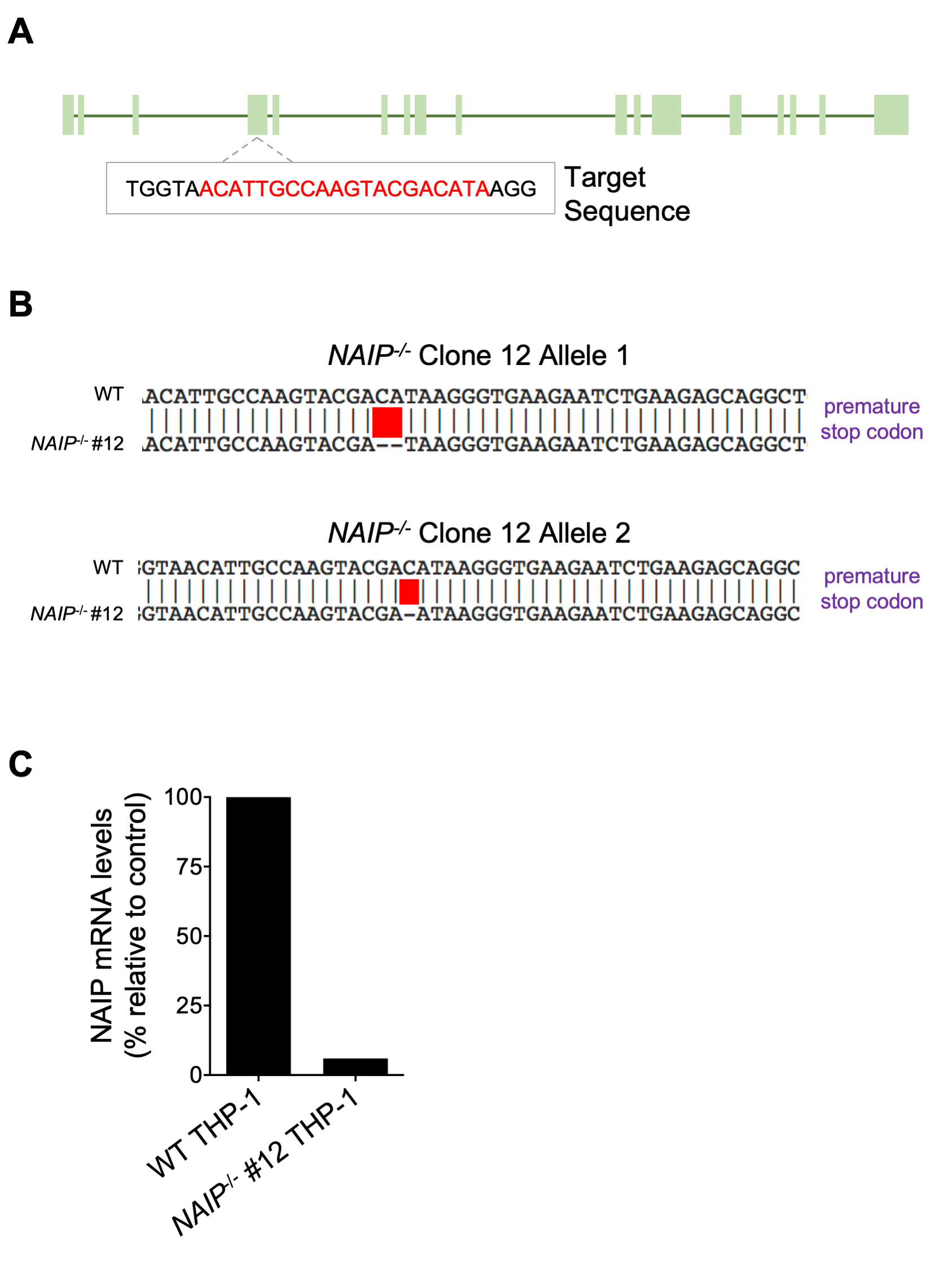

Supplement: S1 Fig — (A) Schematic representation of the NAIP gene with exons (filled boxes) and introns (filled lines). gRNA target sequence is highlighted in red. (B) Sequence alignments of WT THP-1s and NAIP-/- clone #12 are shown for both alleles. Red boxes represent the mutated region. Purple text represents the predicted impact of the mutation on the amino acid sequence. (C) qRT-PCR was performed to quantitate NAIP mRNA levels in WT THP-1s and NAIP-/- THP-1s. For the NAIP-/- THP-1s, NAIP mRNA levels were normalized to human HPRT mRNA levels and WT THP-1s. (TIF) [file ppat.1009718.s001.tif]

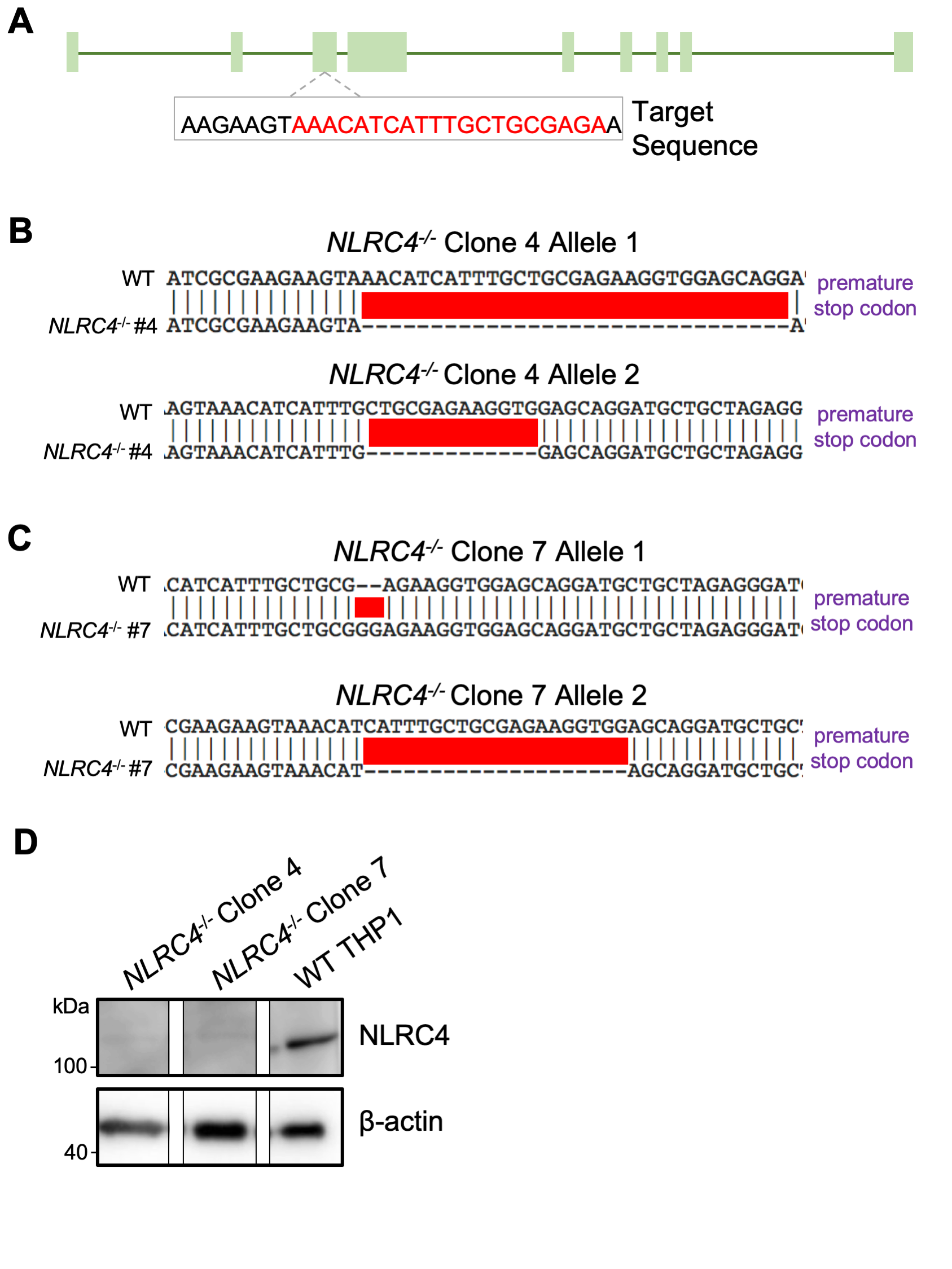

Supplement: S2 Fig — (A) Schematic representation of the NLRC4 gene with exons (filled boxes) and introns (lines). gRNA target sequence is highlighted in red. (B-C) Sequence alignments of WT THP-1s and NLRC4-/- clones are shown for both alleles per clone. Red boxes highlight the mutated region. Purple text represents the predicted impact of the mutation on the amino acid sequence. (D) Immunoblot analysis was performed on cell lysates for human NLRC4, and β-actin as a loading control. (TIF) [file ppat.1009718.s002.tif]

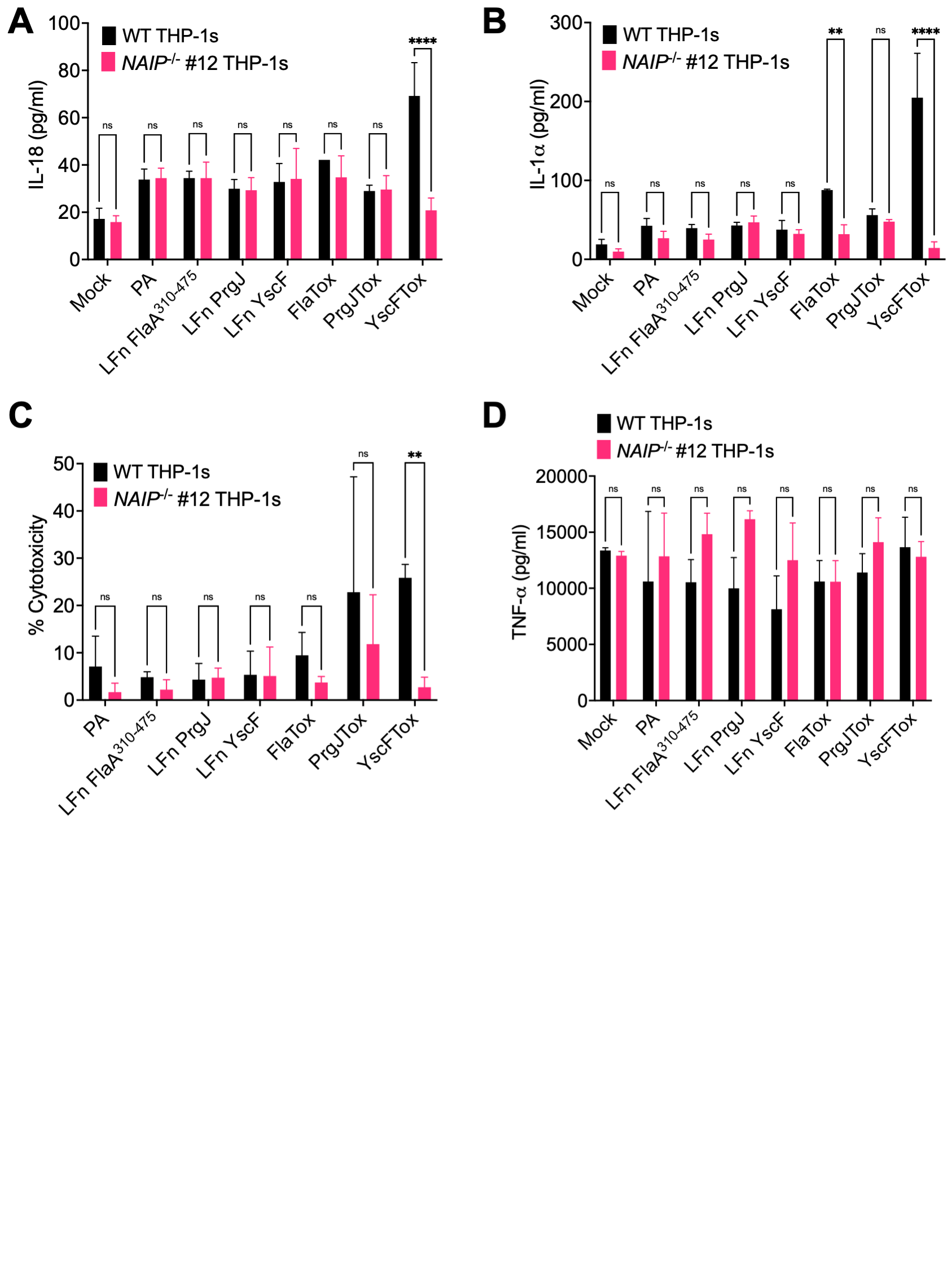

Supplement: S3 Fig — WT or NAIP-/- THP-1 monocyte-derived macrophages were primed with 100 ng/ml Pam3CSK4 for 16 hours. Cells were then treated with PBS (Mock), PA alone, LFn FlaA310–475 (LFn FlaA) alone, LFn PrgJ alone, LF YscF alone, PA+LFn FlaA310–475 (FlaTox), PA+LFn PrgJ (PrgJTox), or PA+LFn YscF (YscFTox) for 6 hours. (A, B, D) Release of cytokines IL-18, IL-1α, and TNF-α into the supernatant were measured by ELISA. (C) Cell death (percentage cytotoxicity) was measured by lactate dehydrogenase release assay and normalized to Mock-treated cells. ns–not significant, **p < 0.01, ****p < 0.0001 by Šídák’s multiple comparisons test. Data shown are representative of at least three independent experiments. (TIF) [file ppat.1009718.s003.tif]

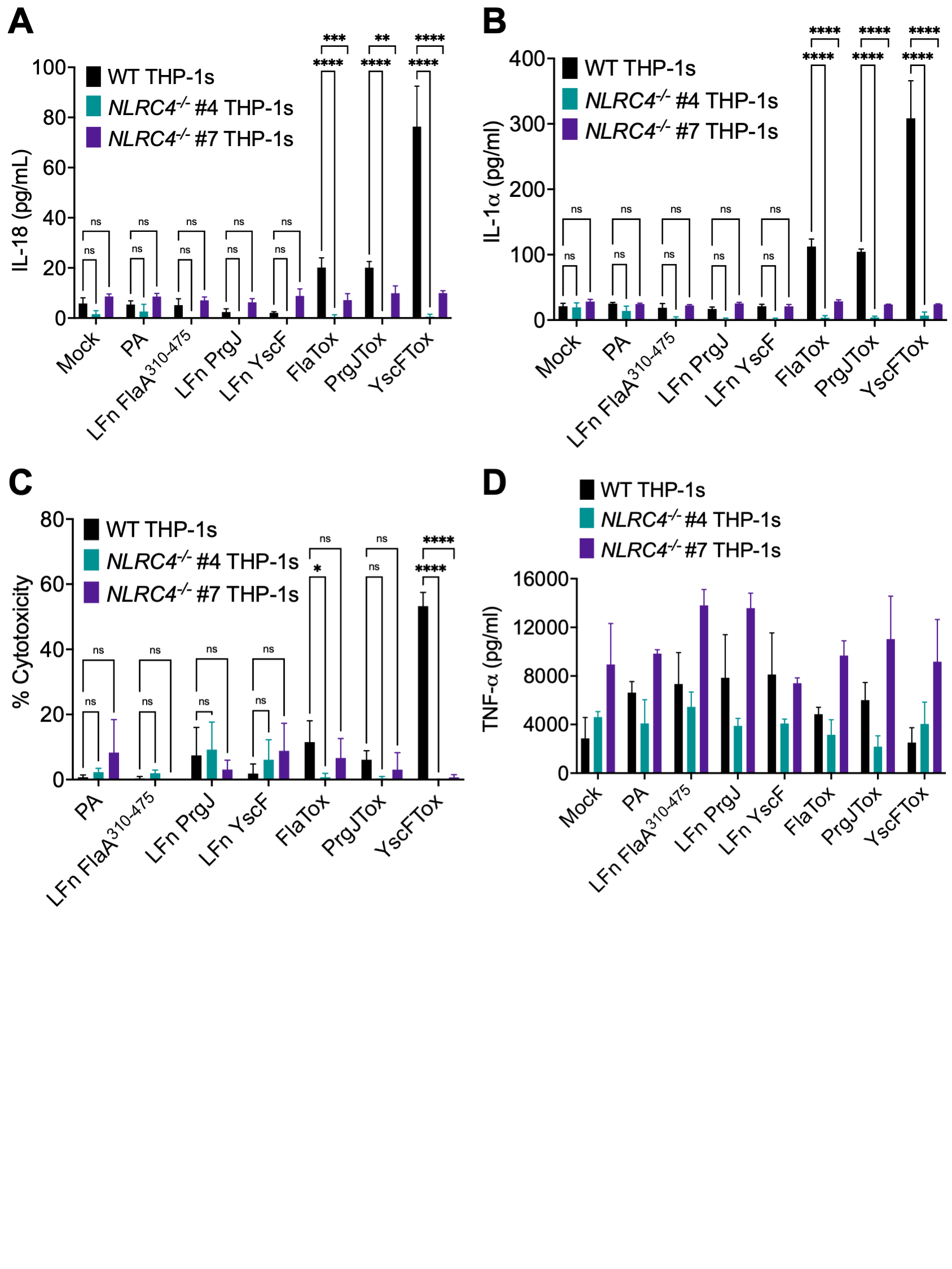

Supplement: S4 Fig — WT or two independent clones of NLRC4-/- THP-1 monocyte-derived macrophages were primed with 100 ng/ml Pam3CSK4 for 16 hours. Cells were then treated with PBS (Mock), PA alone, LFn FlaA310–475 alone, LFn PrgJ alone, LFn YscF alone, PA+LFn FlaA310–475 (FlaTox), PA+LFn PrgJ (PrgJTox), or PA+LFn YscF (YscFTox) for 6 hours. (A, B, D) Release of cytokines IL-18, IL-1α, and TNF-α into the supernatant were measured by ELISA. (C) Cell death (percentage cytotoxicity) was measured by lactate dehydrogenase release assay and normalized to Mock-treated cells. ns–not significant, *p < 0.05, **p < 0.01, ***p < 0.001, ****p < 0.0001 by Dunnett’s multiple comparisons test (A-C). Data shown are representative of at least three independent experiments. (TIF) [file ppat.1009718.s004.tif]

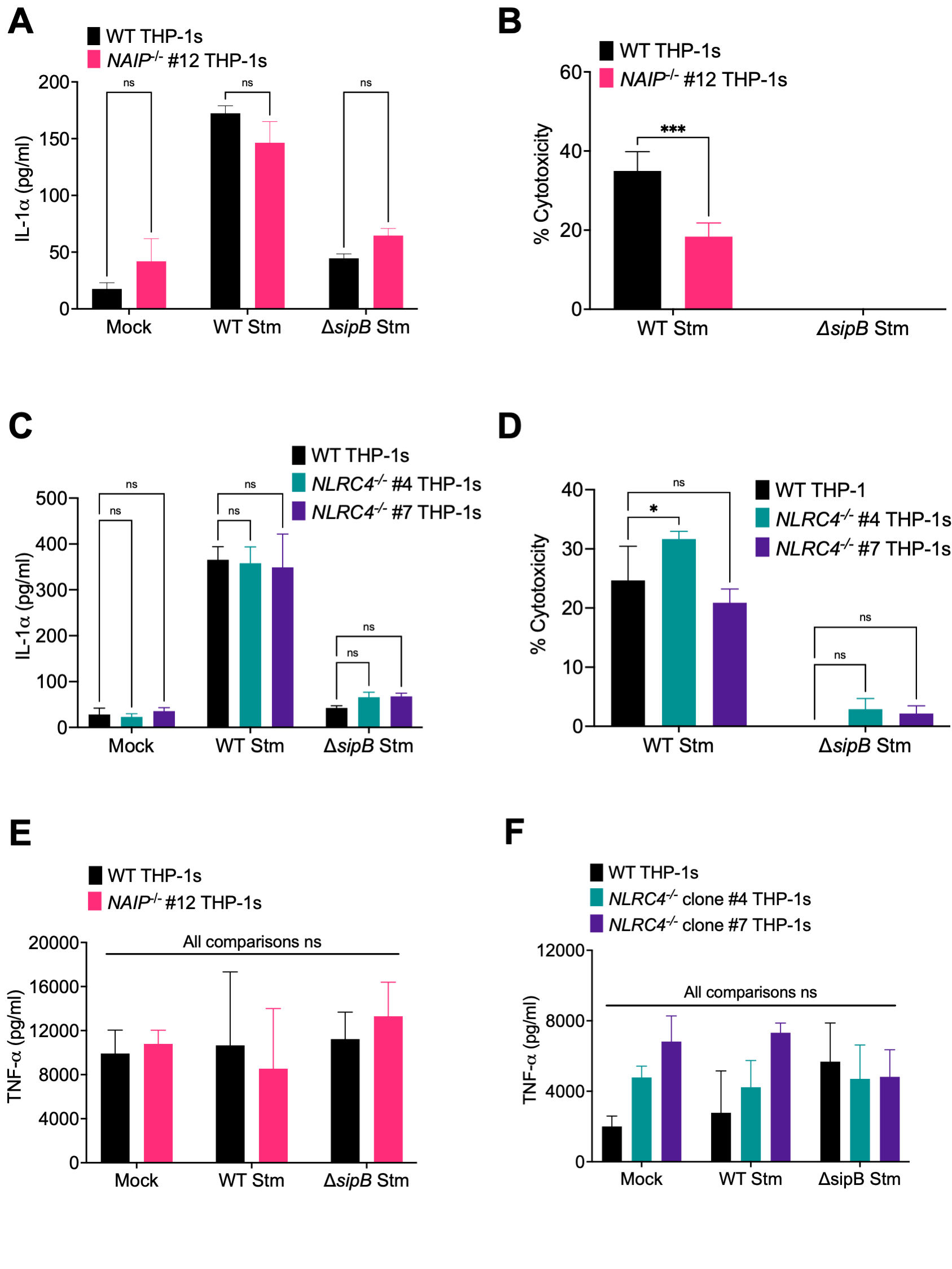

Supplement: S5 Fig — WT, NAIP-/-, or two independent clones of NLRC4-/- THP-1 monocyte-derived macrophages were primed with 100 ng/mL Pam3CSK4 for 16 hours. Cells were then infected with PBS (Mock), WT S. Typhimurium, or ΔsipB S. Typhimurium at an MOI = 20 for 6 hours. As a control, cells were primed with 500 ng/mL LPS for 4 hours and treated with 10 μM nigericin for 6 hours. (A, C, E, F) Release of cytokines IL-1α and TNF-α into the supernatant were measured by ELISA. (B, D) Cell death (percentage cytotoxity) was measured by lactate dehydrogenase release assay and normalized to Mock-treated cells. ns–not significant, *p < 0.05, ***p < 0.001 by Šídák’s multiple comparisons test (A, B, E) or by Dunnett’s multiple comparisons test (C, D, F). Data shown are representative of at least three independent experiments. (TIF) [file ppat.1009718.s005.tif]

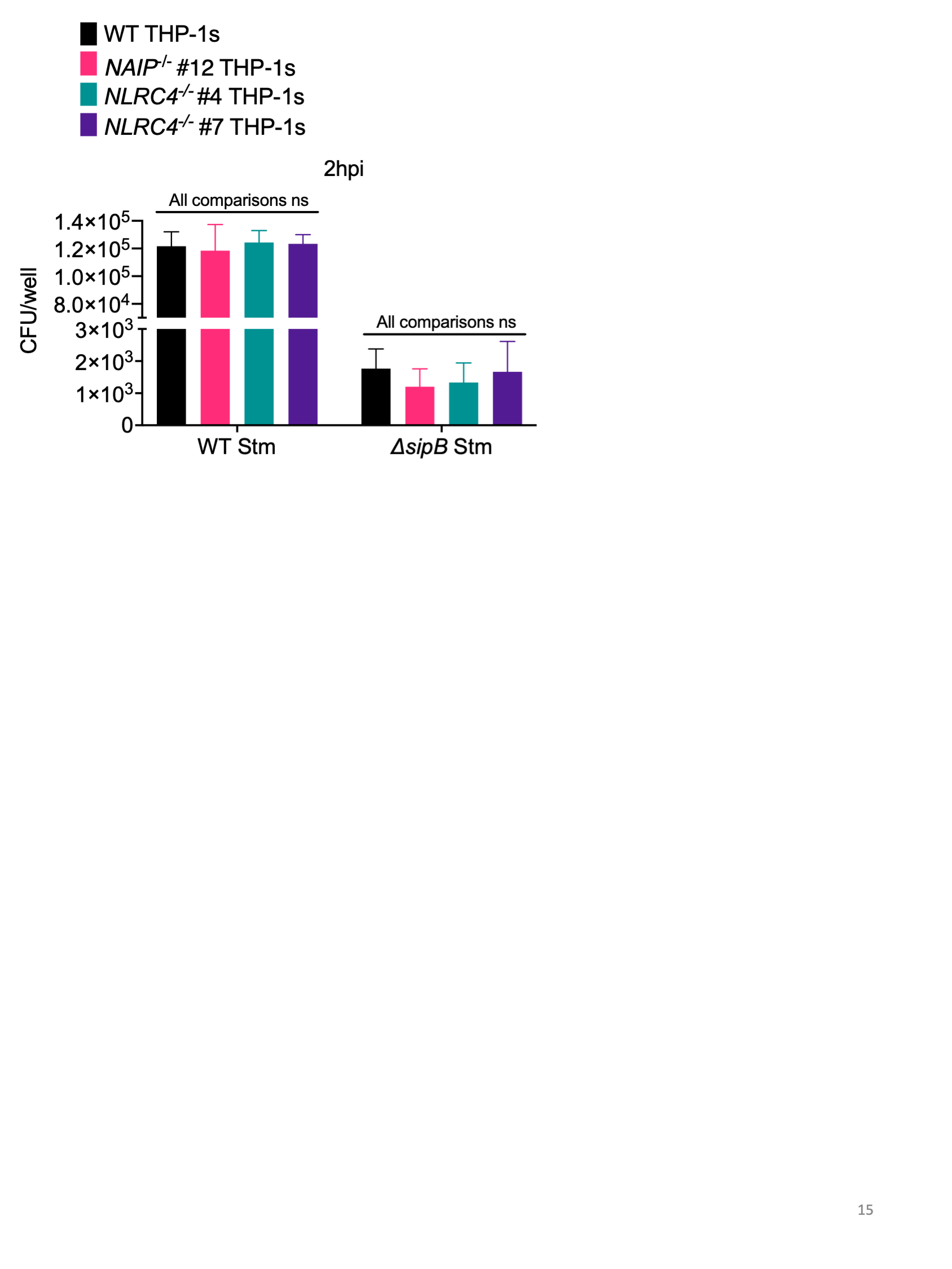

Supplement: S6 Fig — WT, NAIP-/-, and two independent clones of NLRC4-/- THP-1 monocyte-derived macrophages were primed with 100 ng/mL Pam3CSK4 for 16 hours. Cells were then infected with WT S. Typhimurium or ΔsipB S. Typhimurium at an MOI = 20. Cells were lysed at the 2 hours post-infection and bacteria were plated to calculate CFU. ns–not significant, *p < 0.05, ***p < 0.001 by Tukey’s multiple comparisons test. Data shown are representative of at least three independent experiments. (TIF) [file ppat.1009718.s006.tif]

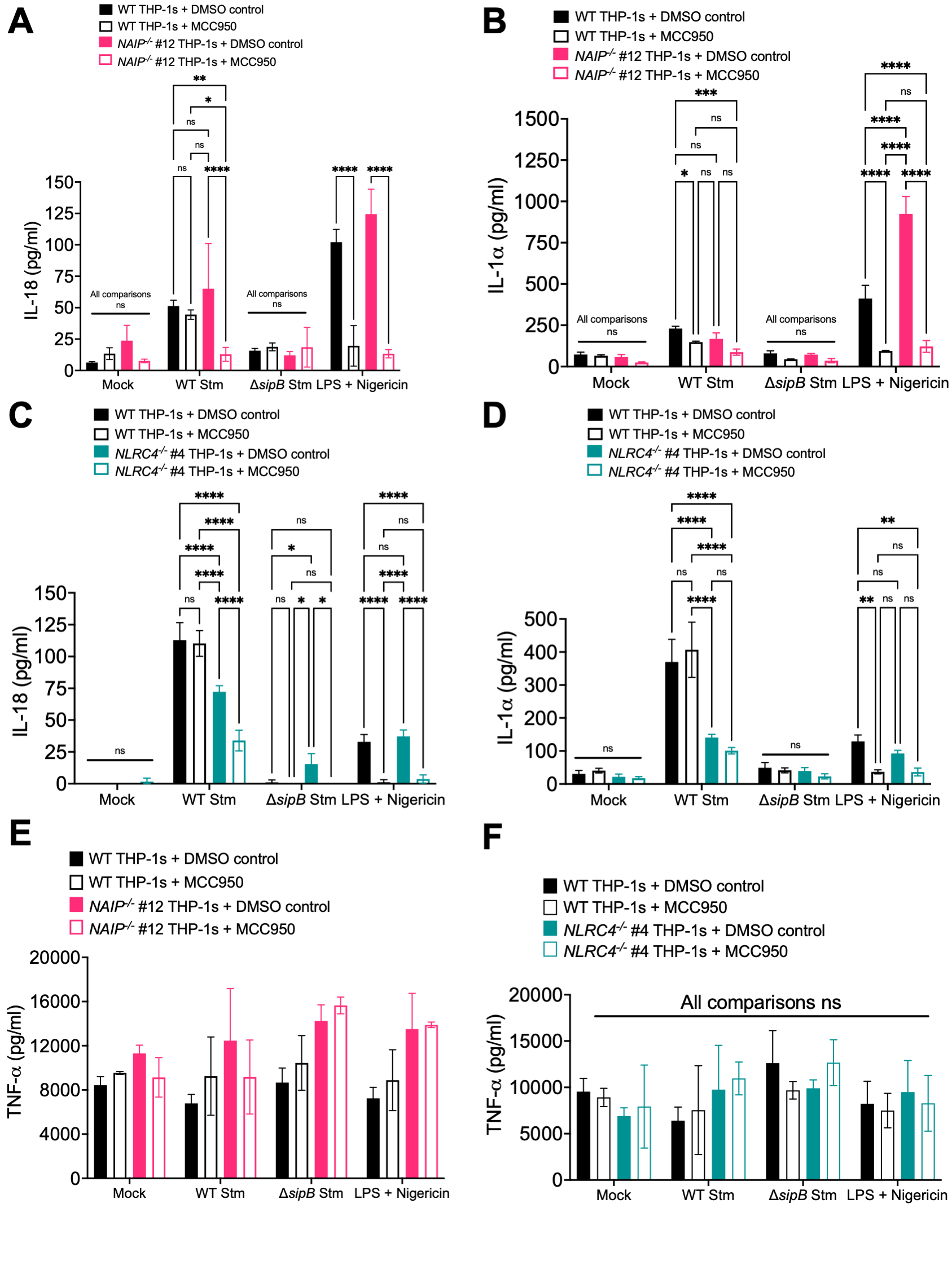

Supplement: S7 Fig — WT, NAIP-/-, or NLRC4-/- THP-1 monocyte-derived macrophages were primed with 100 ng/mL Pam3CSK4 for 16 hours. One hour prior to infection, cells were treated with 1 μM MCC950, a chemical inhibitor of the NLRP3 inflammasome. Cells were then infected with PBS (Mock), WT S. Typhimurium, or ΔsipB S. Typhimurium at an MOI = 20 for 6 hours. (B) As a control, cells were primed with 500 ng/mL LPS for 4 hours and treated with 10 μM nigericin for 6 hours. (A-F) Release of cytokines IL-18, IL-1α, and TNF-α into the supernatant were measured by ELISA. ns–not significant, *p < 0.05, **p < 0.01, ***p < 0.001, ****p < 0.0001 by Tukey’s multiple comparisons test. (TIF) [file ppat.1009718.s007.tif]

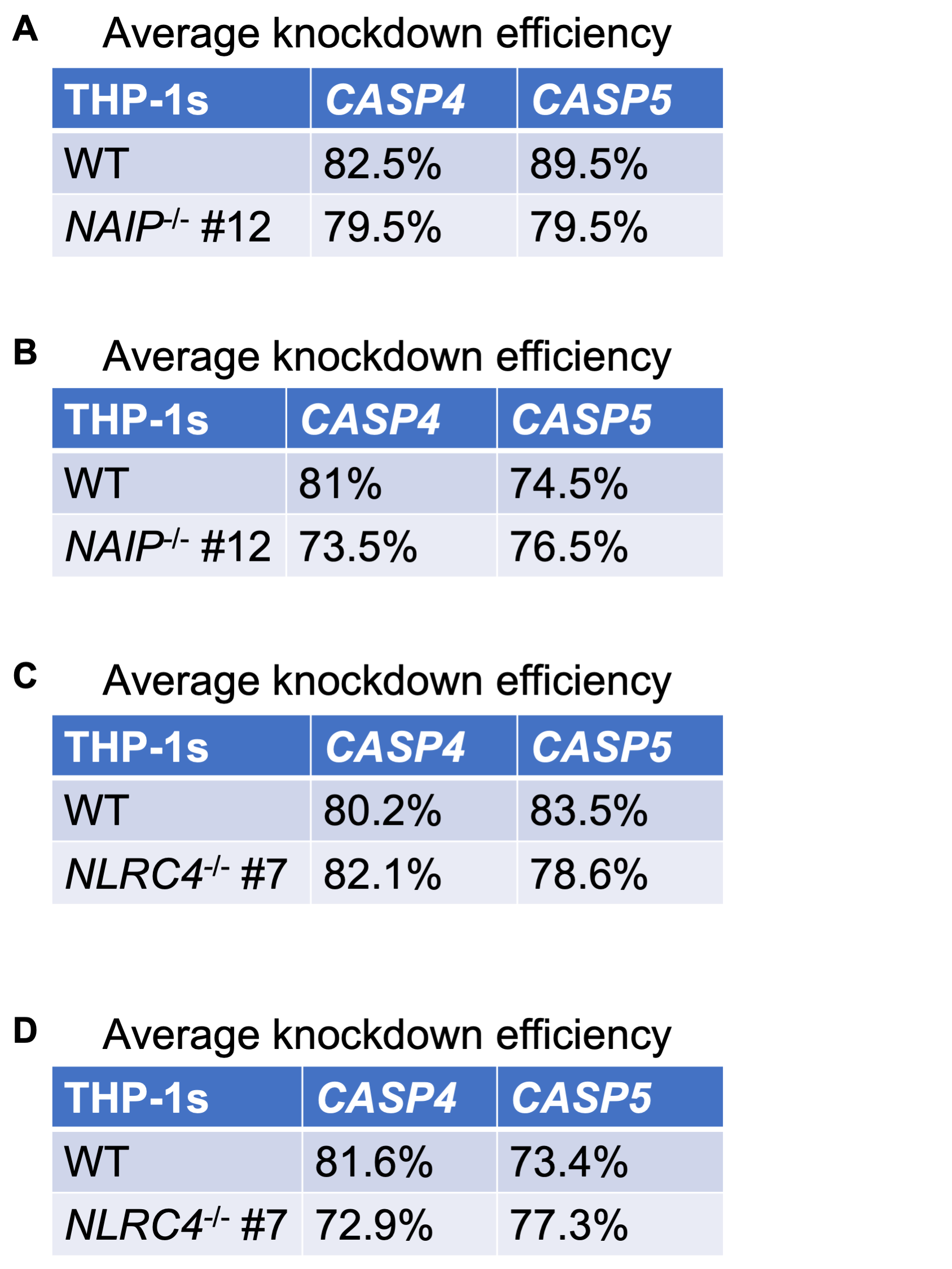

Supplement: S8 Fig — Knockdown efficiencies following siRNA treatment were measured by qRT-PCR and normalized to housekeeping gene HPRT, and calculated relative to control-siRNA-treated cells. (A) siRNA targeting CASP4 or CASP5 in WT vs NAIP-/- #12. (B) siRNA targeting CASP4 and CASP5 in WT vs NAIP-/- #12. (C) siRNA targeting CASP4 or CASP5 in WT vs NLRC4-/- #7. (D) siRNA targeting CASP4 and CASP5 in WT vs NLRC4-/- #7. Data shown are averages of at least three independent experiments. (TIF) [file ppat.1009718.s008.tif]

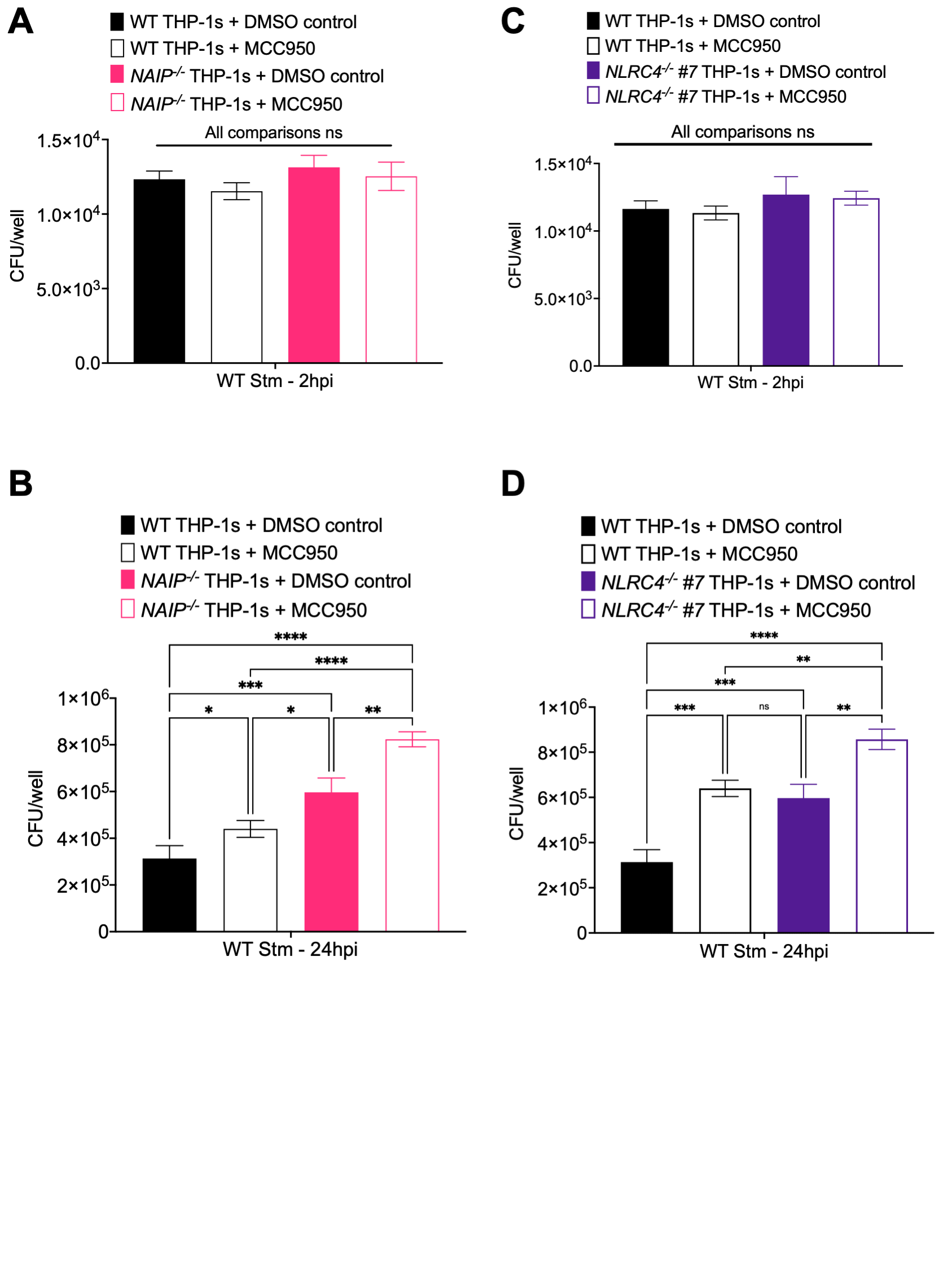

Supplement: S9 Fig — WT, NAIP-/- (A, B), and NLRC4-/- #7 (C, D) THP-1 monocyte-derived macrophages were primed with 100 ng/ml Pam3CSK4 for 16 hours. One hour prior to infection, cells were treated with 1 μM MCC950 or DMSO as a control. Cells were then infected with WT S. Typhimurium at an MOI = 20. Cells were lysed at the indicated time points and bacterial were plated to calculate CFU. (A, C) CFU/well of bacteria at 2 hpi (B, D) CFU/well of bacteria at 24 hpi. *p < 0.05, **p < 0.01, ***p < 0.001, ****p < 0.0001 by Tukey’s multiple comparisons test. Data shown are representative of at least three independent experiments. (TIF) [file ppat.1009718.s009.tif]

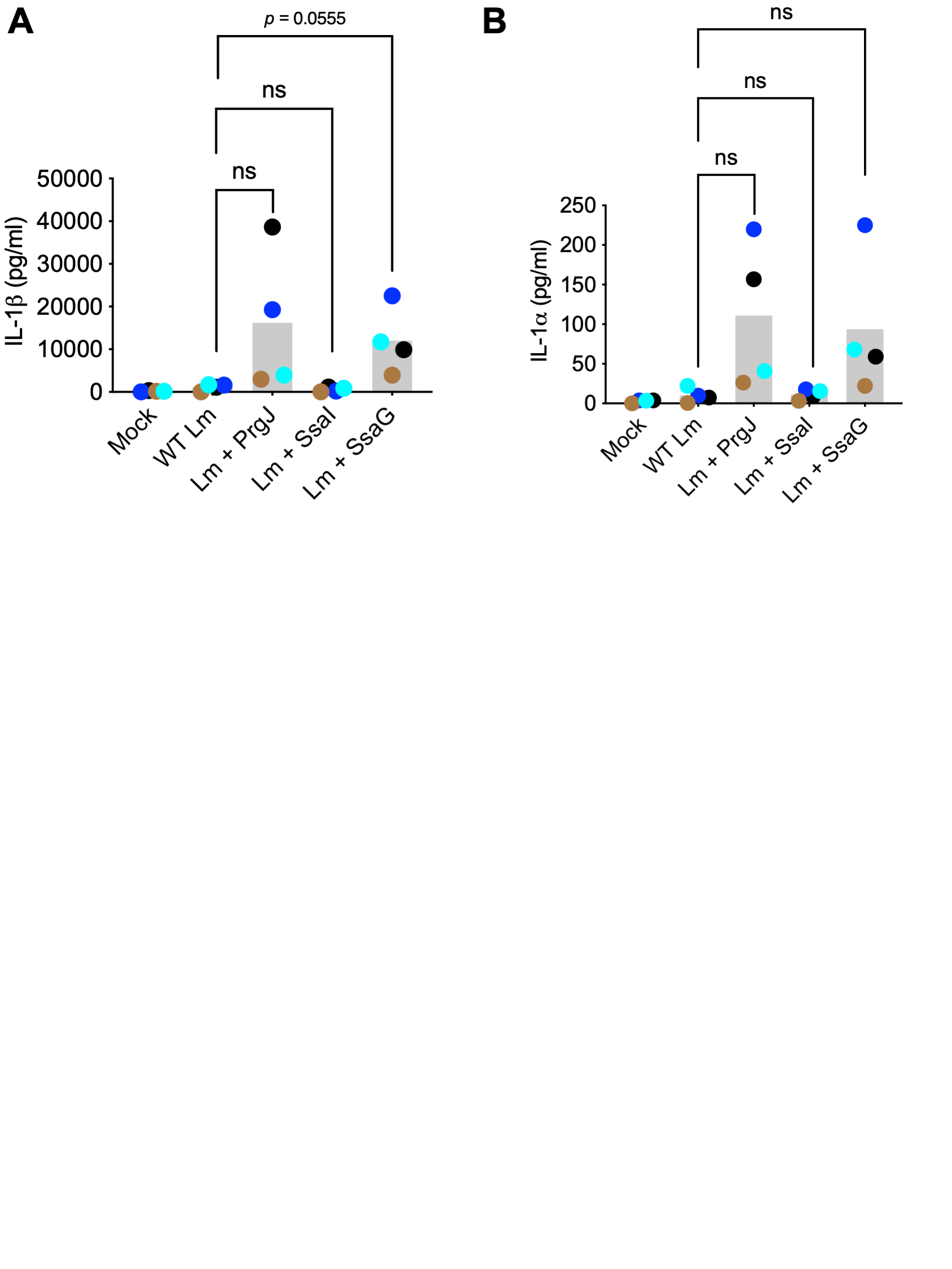

Supplement: S10 Fig — Primary hMDMs from four healthy human donors was infected with PBS (Mock), WT Listeria (WT Lm), Listeria expressing PrgJ (Lm + PrgJ), SsaI (Lm + SsaI), or SsaG (Lm + SsaG) for 16 hours at MOI = 5. Each dot represents the triplicate mean of one donor. The grey bar represents the mean of all donors. Release of cytokines IL-1β and IL-1α was measured by ELISA. p values based on paired t-tests. (TIF) [file ppat.1009718.s010.tif]

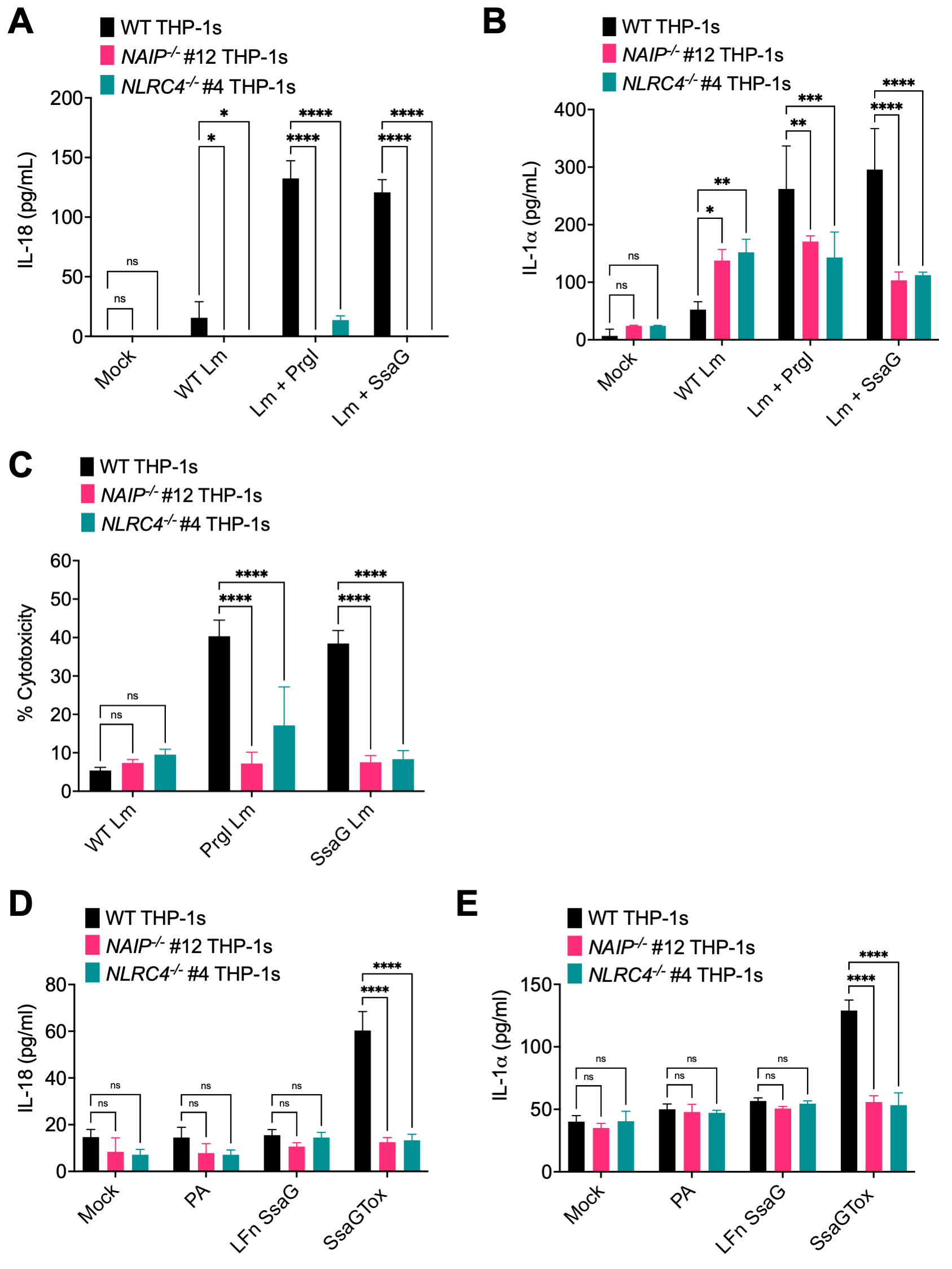

Supplement: S11 Fig — WT, NAIP-/-, or NLRC4-/- THP-1 monocyte-derived macrophages were primed with 100 ng/ml Pam3CSK4 for 16 hours. (A–C) Cells were then treated with PBS (Mock), WT Listeria (WT Lm), Listeria expressing PrgI (Lm + PrgJ) or SsaG (Lm + SsaG) for 6 hours at MOI = 20. (A, B) Release of cytokines IL-18 and IL-1α was measured by ELISA. (C) Cell death was measured by lactate dehydrogenase (LDH) release. (D, E) Cells were treated with PBS (Mock), PA alone, LFn SsaG alone, PA+LFn SsaG (SsaGTox) for 6 hours. Release of cytokines IL-18 and IL-1α was measured by ELISA. ns–not significant, *p < 0.05, **p < 0.01, ***p < 0.001, ****p < 0.0001 by Dunnett’s multiple comparisons test. Data shown are representative of at least three independent experiments. (TIF) [file ppat.1009718.s011.tif]

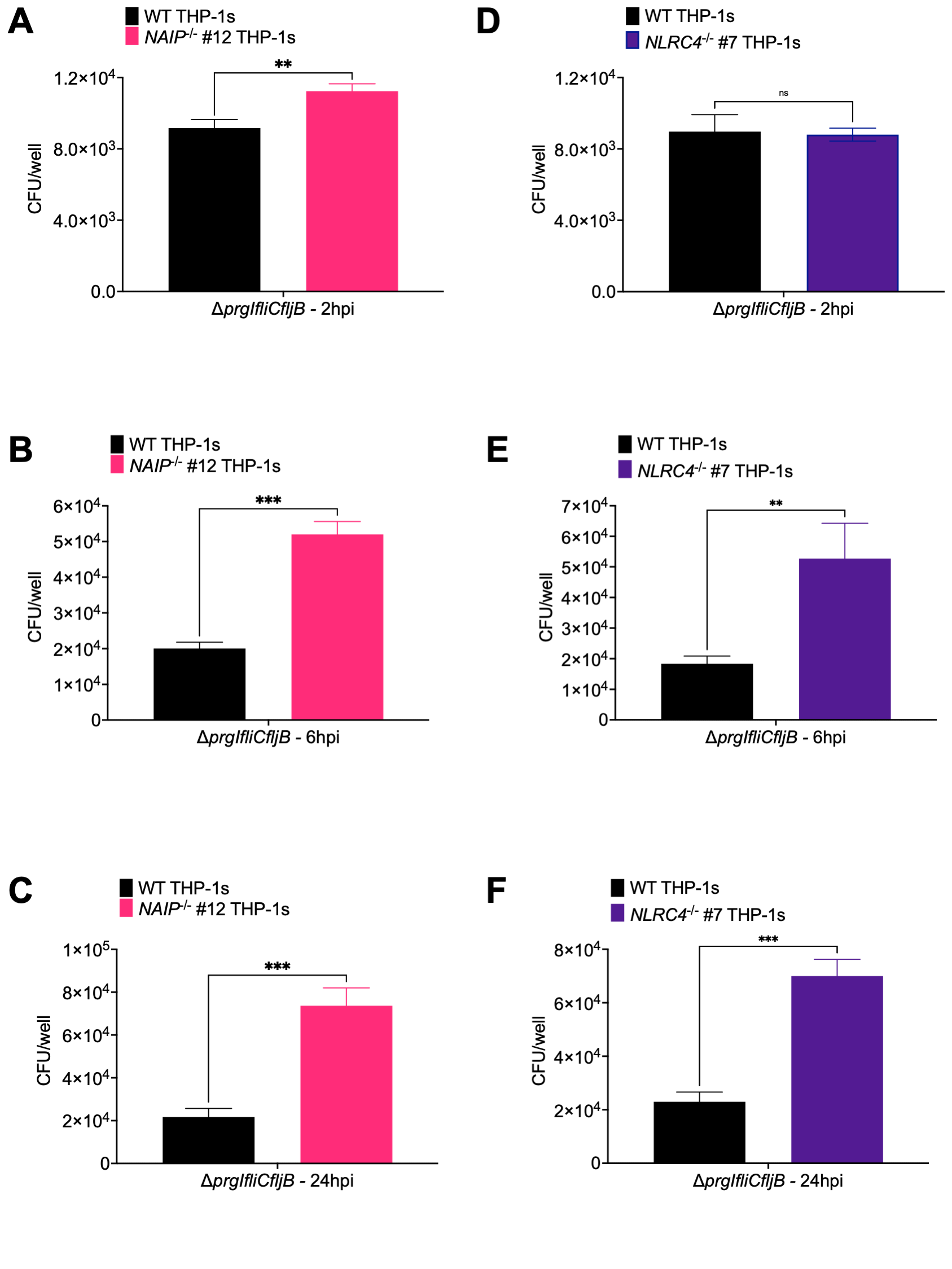

Supplement: S12 Fig — WT, NAIP-/- (A–C) and NLRC4-/- (D–F) THP-1 monocyte-derived macrophages were primed with 100 ng/ml Pam3CSK4 for 16 hours. Cells were then infected with a SPI-1 T3SS/flagellin-deficient strain of S. Typhimurium, ΔprgIfliCfljB at an MOI = 20. (A, D) CFU/well of bacteria at 2 hpi (B, E) CFU/well of bacteria at 6 hpi. (C, F) CFU/well of bacteria at 24 hpi. **p < 0.01, ***p < 0.001, by unpaired t-test. Data shown are representative of at least three independent experiments. (TIF) [file ppat.1009718.s012.tif]

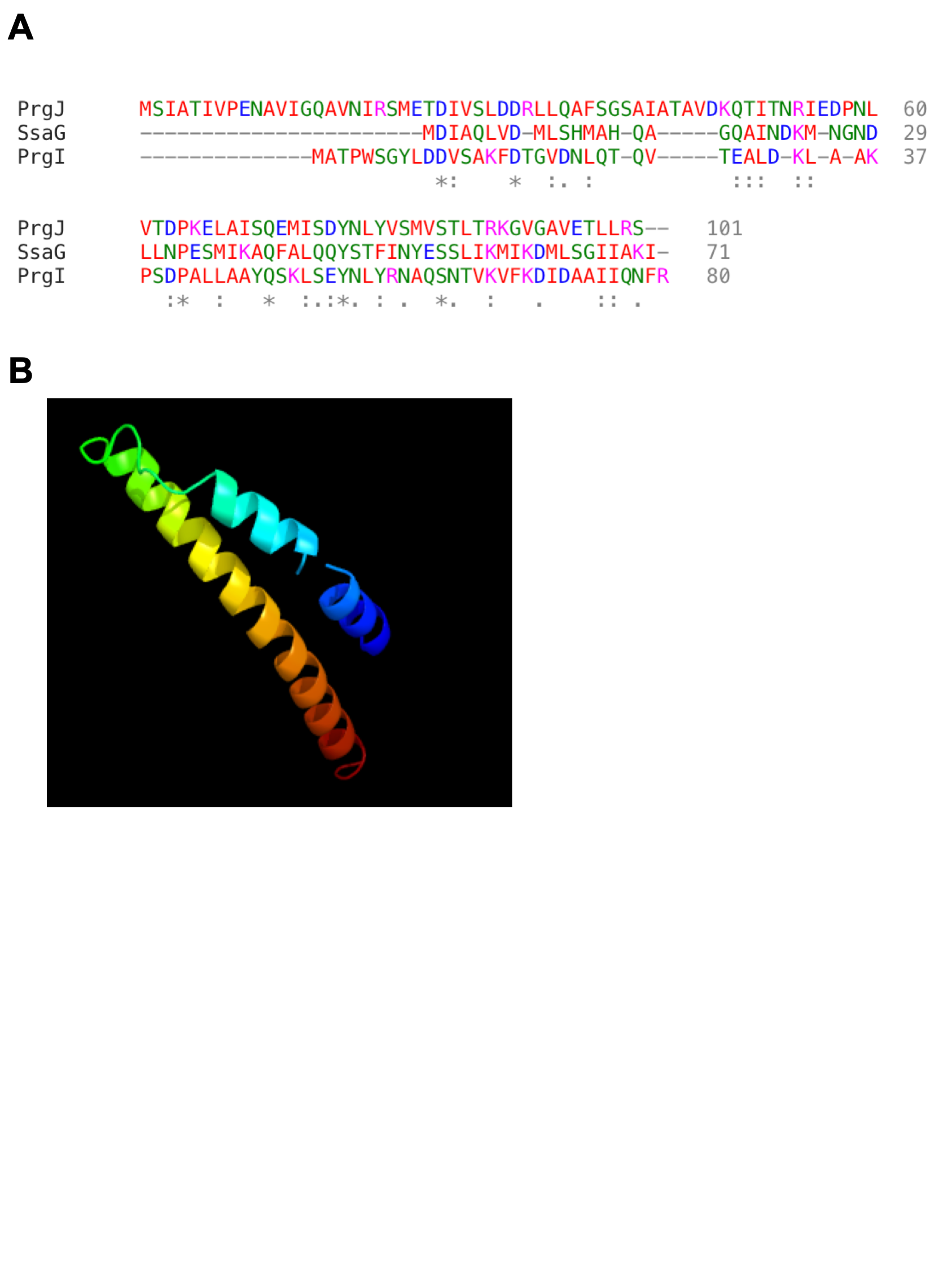

Supplement: S13 Fig — (A) The primary sequences of PrgJ, PrgI, and SsaG were aligned using Multiple Sequence Alignment by Clustal Omega. * indicates single, fully conserved residue,: indicates conservation between groups of strongly similar properties, and. indicates conservation between groups of weakly similar properties. Small, hydrophobic residues are indicated in red (AVFPMILW). Acidic residues are indicated in blue (DE). Basic residues are indicated in magenta (RK). The remaining residues are indicated in green (STYHCNGQ). (B) The three-dimensional structure of SsaG was predicted with high confidence and high coverage using the PHYRE2 server. The structure is colored from N to C terminus using the colors of the rainbow (red, orange, yellow, green, and blue). (TIF) [file ppat.1009718.s013.tif]
