# Supplementary material for: Human NAIP/NLRC4 and NLRP3 inflammasomes detect Salmonella type III secretion system activities to restrict intracellular bacterial replication
Source: PLoS Pathog. 2022 Jan 24;18(1):e1009718. doi: 10.1371/journal.ppat.1009718 (PMC8812861; doi:10.1371/journal.ppat.1009718)
Supplement: S1 File — (DOCX) [file ppat.1009718.s014.docx]

Supplemental Materials and Methods

Generation of CRISPR Cas9 knockouts in THP-1 cells

To knockout *NAIP* and *NLRC4* in THP-1 cells, plasmids encoding the desired guide RNA (gRNA) and Cas9 in the pLentiCRISPR v2 plasmid were purchased from GenScript. The following target sequences were used:

*NAIP*: gRNA 1 (ACATTGCCAAGTACGACATA)

*NLRC4*: gRNA 1 (AAACATCATTTGCTGCGAGA)

For the production of lentiviral particles, pCMV-VSV-G and psPAX2 plasmids were kindly provided by Paul Bates at the University of Pennsylvania. HEK293T cells were plated at 2.5 × 10^6^ cells per 10 cm dish in 10 ml of DMEM supplemented with 10% (vol/vol) heat-inactivated FBS, 2 mM L-glutamine, 100 IU/ml penicillin, and 100 μg/ml streptomycin. After 24 hours, plasmids were transfected using the Lipofectamine 2000 protocol. The amounts of plasmids used were 1 μg of pCMV-VSV-G, 2.5 μg of psPAX2, and 8 μg of pLentiCRISPR v2 encoding the appropriate gRNA. 50 μl of Lipofectamine 2000 was used per dish. Transfected HEK293T cells were incubated for 18 hours at 37°C, and the media was then aspirated and replaced with 6 ml of fresh growth media. After 16 - 18 hours, the supernatant containing lentiviral particles was harvested and filtered using a 0.22 μM filter. THP-1 cells were infected in 1 ml of viral-containing media with 8 μg/ml of polybrene. The cells were spin-infected at 1250 ́× *g* for 90 min at 25°C, then incubated at 37°C for 48 hours. After 48 hours, puromycin was added to a final concentration of 1 μg/ml. The cells were maintained in puromycin for 3 weeks and then harvested for Western Blot analysis and clonal selection. For clonal selection, cells were plated in 96-well plates at 0.5 cell per well or 2 cells per well in 200 μl of growth media and were incubated for 4-8 weeks until single clones were visible in the bottom of the well. Single cell clones were then expanded from a 96-well plate through a series of multi-well plates ending in 10 cm dishes. Cells were then plated in 48-well plates at a concentration of 2.0 × 10^5^ cells per well in 500 μl of media and harvested for purification of RNA, DNA, and Western Blot assays.

Validation of CRISPR Cas9 THP-1 single clones for knockouts

To validate single cell clones after CRISPR Cas9 editing, DNA was purified using the DNeasy Blood and Tissue kit (Qiagen). The genomic region containing the target sequence was then amplified by PCR using the following primers (all 5’ to 3’):

*NAIP* forward: CCGTACAGCTCATGGATACCACAG

*NAIP* reverse: GTACCTGTAAAGACAAAGCCAGCC

*NLRC4* forward: CCCAGCCGGATATGCACATT

*NLRC4* reverse: TCTGCCATGGGGAAGATGGAT

The PCR product was purified using the PCR Cleanup kit (Qiagen). 3’ A overhangs were added to the purified PCR product by combining 7 μl of PCR product, 5 Units of Taq DNA polymerase, 1X PCR Buffer containing MgCl_2_, and 0.2mM dATP. The reaction was then incubated at 70°C for 30 minutes. 2 μl of this product was then ligated into the pGEM-T vector and transformed into DH5$\alpha$ competent cells using the protocol in the pGEM-T Vector System Protocol (A1360; Promega). Positive colonies were sequenced using a T7 Promoter Primer (5′ TAATACGACTCACTATAGGG 3′).

Quantitative RT-PCR Analysis of *NAIP^-/-^* THP-1s

Cells were lysed and RNA was isolated using the RNeasy Plus Kit (Qiagen). Synthesis of the first strand cDNA was performed using Superscript II reverse transcriptase and oligo (dT) primer (Invitrogen). Quantitative PCR was performed with the CFX96 real-time system (Bio-Rad) using the SsoFast EvaGreen Supermix with LOW ROX kit (Bio-Rad). The following primers were used (all 5’-3’):

*NAIP* forward: GCATTCTCCTCTATTAGACTAG

*NAIP* reverse: GCCAACTGAACTGCATCTAG

*HPRT* forward: CCTGGCGTCGTGATTAGTGAT

*HPRT* reverse: AGACGTTCAGTCCTGTCCATAA

For analysis, mRNA levels of CRISPR-modified THP-1 cells were normalized to control THP-1 cells using the 2^−ΔΔCT^ (cycle threshold) method [1] to calculate relative expression.

Immunoblotting of *NLRC4*^-/-^ THP-1s

Cells were lysed directly with 1X SDS-PAGE sample buffer. Protein samples were boiled for 5 minutes, separated by SDS-PAGE, and transferred to a PVDF Immobilon-P membranes (Millipore). Samples were then probed with antibody specific for NLRC4 (12421S; Cell Signaling). As a loading control, the blot was probed with anti-β-actin (4967L; Cell Signaling). Detection was performed with HRP-conjugated anti-mouse IgG (F00011; Cell Signaling) or anti-rabbit IgG (7074S; Cell Signaling).

Three-dimensional structure prediction using PHYRE2

The PHYRE2 server was used for protein modeling, prediction, and analysis of the published protein sequence for SsaG from *Salmonella* Typhimurium SL1344. The sequence is shown below (N terminus to C terminus:

MDIAQLVDMLSHMAHQAGQAINDKMNGNDLLNPESMIKAQFALQQYSTFINYESSLIKMIKDMLSGIIAKI

PHYRE2 is a bioinformatics tool that uses homology-based modeling to predict the three-dimensional structure of proteins [2,3]. For the structural modeling of SsaG, the confidence score was 98% and the coverage was 96%. Thus, the structure predicted for SsaG is considered to be highly confident with high coverage.

Supplemental References

1. Livak KJ, Schmittgen TD. Analysis of Relative Gene Expression Data Using Real-Time Quantitative PCR and the 2−ΔΔCT Method. Methods. 2001;25: 402–408. doi:10.1006/meth.2001.1262

2. Kelley LA, Mezulis S, Yates CM, Wass MN, Sternberg MJE. The Phyre2 web portal for protein modeling, prediction and analysis. Nat Protoc. 2015;10: 845–858. doi:10.1038/nprot.2015.053

3. Phyre2: Protein Homology/analogy Recognition Engine V 2.0. Available: http://www.sbg.bio.ic.ac.uk/phyre2.

Supplemental Figure Legends

**S1 File**. **Supplemental Materials and Methods**.

Materials and methods for the supplemental figures are listed.

**S1 Fig. Validation of *NAIP* mutant THP-1 single cell clones generated with CRISPR/Cas9 genome editing.**

(A) Schematic representation of the *NAIP* gene with exons (filled boxes) and introns (filled lines). gRNA target sequence is highlighted in red. (B) Sequence alignments of WT THP-1 and *NAIP^-/-^* clone 12 are shown for both alleles. Red boxes represent the mutated region. Purple text represents the predicted impact of the mutation on the amino acid sequence. (C) qRT-PCR was performed to quantitate *NAIP* mRNA levels in WT THP-1 and *NAIP^-/-^* THP-1s. For the *NAIP^-/-^* THP-1s*, NAIP* mRNA levels were normalized to human HPRT mRNA levels and WT THP-1s.

**S2 Fig. Validation of *NLRC4* mutant THP-1 single cell clones generated with CRISPR/Cas9-mediated genome editing.**

(A) Schematic representation of the *NLRC4* gene with exons (filled boxes) and introns (lines). gRNA target sequence is highlighted in red. (B-C) Sequence alignments of WT THP-1 and *NLRC4^-/-^* clones are shown for both alleles per clone. Red boxes highlight the mutated region. Purple text represents the predicted impact of the mutation on the amino acid sequence. (D) Immunoblot analysis was performed on cell lysates for human NLRC4, and β-actin as a loading control.

**S3 Fig. (related to Fig 1) NAIP is necessary for inflammasome responses to T3SS ligands in human macrophages.**

WT or *NAIP^-/-^* THP-1 monocyte-derived macrophages were primed with 100 ng/ml Pam3CSK4 for 16 hours. Cells were then treated with PBS (Mock), PA alone, LFn FlaA^310–475^ alone, LFn PrgJ alone, LF YscF alone, PA+LFn FlaA^310–475^ (FlaTox), PA+LFn PrgJ (PrgJTox), or PA+LFn YscF (YscFTox) for 6 hours. (A, B, D) Release of cytokines IL-18, IL-1α, and TNF-α into the supernatant were measured by ELISA. (C) Cell death (percentage cytotoxicity) was measured by lactate dehydrogenase release assay and normalized to Mock-treated cells. ns – not significant, ***p* < 0.01, *****p* < 0.0001 by Šídák’s multiple comparisons test. Data shown are representative of at least three independent experiments.

**S4 Fig. (related to Fig 1) NLRC4 is necessary for inflammasome responses to T3SS ligands in human macrophages.**

WT or two independent clones of *NLRC4^-/-^* THP-1 monocyte-derived macrophages were primed with 100 ng/ml Pam3CSK4 for 16 hours. Cells were then treated with PBS (Mock), PA alone, LFn FlaA^310–475^ alone, LFn PrgJ alone, LFn YscF alone, PA+LFn FlaA^310–475^ (FlaTox), PA+LFn PrgJ (PrgJTox), or PA+LFn YscF (YscFTox) for 6 hours. (A, B, D) Release of cytokines IL-18, IL-1α, and TNF-α into the supernatant were measured by ELISA. (C) Cell death (percentage cytotoxicity) was measured by lactate dehydrogenase release assay and normalized to Mock-treated cells. ns – not significant, **p* < 0.05, ***p* < 0.01, ****p* < 0.001, *****p* < 0.0001 by Dunnett’s multiple comparisons test (A-C). Data shown are representative of at least three independent experiments.

**S5 Fig. (related to Fig 2) NAIP and NLRC4 are partially required for inflammasome activation during *Salmonella* infection in human macrophages.**

WT, *NAIP^-/-^,* or two independent clones of *NLRC4^-/-^* THP-1 monocyte-derived macrophages were primed with 100 ng/uL Pam3CSK4 for 16 hours. Cells were then infected with PBS (Mock), WT *S*. Typhimurium, or Δ*sipB* *S*. Typhimurium at an MOI=20 for 6 hours. As a control, cells were primed with 500 ng/mL LPS for 4 hours and treated with 10 uM nigericin for 6 hours. (A, C, E, F) Release of cytokines IL-1α and TNF-α into the supernatant were measured by ELISA. (B, D) Cell death (percentage cytotoxity) was measured by lactate dehydrogenase release assay and normalized to Mock-treated cells. ns – not significant, **p* < 0.05, ****p* < 0.001 by Šídák’s multiple comparisons test (A, B, E) or by Dunnett’s multiple comparisons test (C, D, F). Data shown are representative of at least three independent experiments.

**S6 Fig (related to Fig 2). Uptake of *Salmonella* into THP-1 macrophages.**

WT, *NAIP^-/-^,* and two independent clones of *NLRC4^-/-^* THP-1 monocyte-derived macrophages were primed with 100 ng/uL Pam3CSK4 for 16 hours. Cells were then infected with WT *S*. Typhimurium or Δ*sipB* *S*. Typhimurium at an MOI=20. Cells were lysed at the 2 hours post-infection and bacteria were plated to calculate CFU. ns – not significant, **p* < 0.05, ****p* < 0.001 by Tukey’s multiple comparisons test. Data shown are representative of at least three independent experiments.

**S7 Fig. (related to Fig 3) *Salmonella* induces NAIP/NLRC4- and NLRP3-dependent inflammasome activation in human macrophages.**

WT, *NAIP^-/-^,* or *NLRC4^-/-^* THP-1 monocyte-derived macrophages were primed with 100 ng/uL Pam3CSK4 for 16 hours. One hour prior to infection, cells were treated with 1 µM MCC950, a chemical inhibitor of the NLRP3 inflammasome. Cells were then infected with PBS (Mock), WT *S*. Typhimurium, or Δ*sipB* *S*. Typhimurium at an MOI=20 for 6 hours. (B) As a control, cells were primed with 500 ng/mL LPS for 4 hours and treated with 10 uM nigericin for 6 hours. (A-F) Release of cytokines IL-18, IL-1α, TNF-α into the supernatant were measured by ELISA. ns – not significant, **p* < 0.05, ***p* < 0.01, ****p* < 0.001, *****p* < 0.0001 by Tukey’s multiple comparisons test.

**S8 Fig. (related to Fig 4) Knockdown efficiencies of siRNA-mediated silencing of *CASP4* and *CASP5* in human macrophages.**

Knockdown efficiencies following siRNA treatment were measured by qRT-PCR and normalized to housekeeping gene *HPRT*, and calculated relative to control-siRNA-treated cells. (A) siRNA targeting *CASP4* or *CASP5* in WT vs *NAIP^-/-^* #12. (B) siRNA targeting *CASP4* and *CASP5* in WT vs *NAIP^-/-^* #12. (C) siRNA targeting *CASP4* or *CASP5* in WT vs *NLRC4^-/-^* #7. (D) siRNA targeting *CASP4* and *CASP5* in WT vs *NLRC4^-/-^* #7. Data shown are averages of at least three independent experiments.

**S9 Fig. (related to Fig 5) NAIP and NLRP3 restrict replication of *Salmonella* in human macrophages.**

WT, *NAIP^-/-^* (A, B), and *NLRC4^-/-^* #7 (C, D) THP-1 monocyte-derived macrophages were primed with 100 ng/ml Pam3CSK4 for 16 hours. One hour prior to infection, cells were treated with 1 µM MCC950 or DMSO as a control. Cells were then infected with WT *S*. Typhimurium at an MOI=20. Cells were lysed at the indicated time points and bacterial were plated to calculate CFU. (A, C) CFU/well of bacteria at 2 hpi (B, D) CFU/well of bacteria at 24 hpi. **p* < 0.05, ***p* < 0.01, ****p* < 0.001, *****p* < 0.0001 by Tukey’s multiple comparisons test. Data shown are representative of at least three independent experiments.

**S10 Fig. (related to Fig 7A) *Salmonella* SPI-2 needle protein SsaG activates the inflammasome in human macrophages.**

Primary hMDMs from four healthy human donors was infected with PBS (Mock), WT *Listeria* (WT Lm), *Listeria* expressing PrgJ (Lm + PrgJ), SsaI (Lm + SsaI), or SsaG (Lm + SsaG) for 16 hours at MOI=5. Each dot represents the triplicate mean of one donor. The grey bar represents the mean of all donors. Release of cytokines IL-1β and IL-1α, was measured by ELISA. *p* values based on paired t-tests.

**S11 Fig. (related to Fig 7B and 7C) NAIP/NLRC4 are necessary for inflammasome responses to the *Salmonella* SPI-2 needle protein SsaG in human macrophages.**

WT, *NAIP^-/-^*, or *NLRC4^-/-^* THP-1 monocyte-derived macrophages were primed with 100 ng/ml Pam3CSK4 for 16 hours. (A – C) Cells were then treated with PBS (Mock), WT *Listeria* (WT Lm), *Listeria* expressing PrgI (Lm + PrgJ) or SsaG (Lm + SsaG) for 6 hours at MOI=20. (A, B) Release of cytokines IL-18, and IL-1α was measured by ELISA. (C) Cell death was measured by lactate dehydrogenase (LDH) release. (D, E) Cells were treated with PBS (Mock), PA alone, LFn SsaG alone, PA+LFn SsaG (SsaGTox) for 6 hours. Release of cytokines IL-18, and IL-1α was measured by ELISA. ns – not significant, **p* < 0.05, ***p* < 0.01, ****p* < 0.001, *****p* < 0.0001 by Dunnett’s multiple comparisons test. Data shown are representative of at least three independent experiments.

**S12 Fig. (related to Fig 8) NAIP/NLRC4 inflammasome recognition of the SPI-2 T3SS restricts *Salmonella* replication in human macrophages.**

WT, *NAIP^-/-^* (A – C) and *NLRC4^-/-^* (D – F) THP-1 monocyte-derived macrophages were primed with 100 ng/ml Pam3CSK4 for 16 hours. Cells were then infected with a SPI-1 T3SS/flagellin-deficient strain of *S*. Typhimurium, Δ*prgIfliCfljB* at an MOI=20. (A, D) CFU/well of bacteria at 2 hpi (B, E) CFU/well of bacteria at 6 hpi. (C, F) CFU/well of bacteria at 24 hpi. ***p* < 0.01, ****p* < 0.001, by unpaired t-test. Data shown are representative of at least three independent experiments.

**S13 Fig. Sequence alignment and three-dimensional structural prediction of SsaG.**

(A) The primary sequences of PrgJ, PrgI, and SsaG were aligned using Multiple Sequence Alignment by Clustal Omega. ***** indicates single, *fully conserved* residue, **:** indicates conservation between groups of *strongly* similar properties, and **.** indicates conservation between groups of *weakly* similar properties. Small, hydrophobic residues are indicated in red (AVFPMILW). Acidic residues are indicated in blue (DE). Basic residues are indicated in magenta (RK). The remaining residues are indicated in green (STYHCNGQ). (B) The three-dimensional structure of SsaG was predicted with high confidence and high coverage using the PHYRE2 server. The structure is colored from N to C terminus using the colors of the rainbow (red, orange, yellow, green, and blue).
